# EBV–encoded miRNAs can sensitize nasopharyngeal carcinoma to chemotherapeutic drugs by targeting BRCA1

**DOI:** 10.1111/jcmm.16007

**Published:** 2020-10-19

**Authors:** Raymond Wai‐Ming Lung, Joanna Hung‐Man Tong, Lok‐Man Ip, Ka‐Hei Lam, Anthony Wing‐Hung Chan, Wing‐Po Chak, Lau‐Ying Chung, Walter Wai Yeung, Pok‐Man Hau, Shuk‐Ling Chau, Sai‐Wah Tsao, Kin‐Mang Lau, Kwok‐Wai Lo, Ka‐Fai To

**Affiliations:** ^1^ Department of Anatomical and Cellular Pathology State Key Laboratory of Translational Oncology Sir Y.K. Pao Cancer Center Prince of Wales Hospital The Chinese University of Hong Kong Hong Kong China; ^2^ Department of Biomedical Sciences and Center of Nasopharyngeal Carcinoma Research Li Ka Shing Faculty of Medicine The University of Hong Kong Pokfulam China; ^3^ Institute of Digestive Disease Partner State Key Laboratory of Digestive Disease The Chinese University of Hong Kong Hong Kong China

**Keywords:** BRCA1, Epstein‐Barr virus, *miR‐BARTs*, nasopharyngeal carcinoma

## Abstract

Nasopharyngeal carcinoma (NPC) is an Epstein‐Barr virus (EBV)‐associated epithelial malignancy. The high expression of *BART*‐miRNAs (*miR‐BARTs*) during latent EBV infection in NPC strongly supports their pathological importance in cancer progression. Recently, we found that several *BART*‐miRNAs work co‐operatively to modulate the DNA damage response (DDR) by reducing Ataxia‐telangiectasia‐mutated (ATM) activity. In this study, we further investigated the role of *miR‐BARTs* on DDR. The immunohistochemical study showed that the DNA repair gene, *BRCA1*, is consistently down‐regulated in primary NPCs. Using computer prediction programs and a series of reporter assays, we subsequently identified the negative regulatory role of *BART2‐3p*, *BART12*, *BART17‐5p* and *BART19‐3p* in BRCA1 expression. The ectopic expression of these four *miR‐BARTs* suppressed endogenous BRCA1 expression in EBV‐negative epithelial cell lines, whereas BRCA1 expression was enhanced by repressing endogenous *miR‐BARTs* activities in C666‐1 cells. More importantly, suppressing BRCA1 expression in nasopharyngeal epithelial cell lines using *miR‐BART17‐5p* and *miR‐BART19‐3p* mimics reduced the DNA repair capability and increased the cell sensitivity to the DNA‐damaging chemotherapeutic drugs, cisplatin and doxorubicin. Our findings suggest that *miR‐BARTs* play a novel role in DDR and may facilitate the development of effective NPC therapies.

## 1 INTRODUCTION

Nasopharyngeal carcinoma (NPC) is an aggressive epithelial malignancy that originates in the nasopharynx; it is prevalent in Southeast Asia. Due to the advancement of diagnostic and therapeutic strategies, the mortality rate of NPC in Hong Kong has continuously declined, falling from 8.6 per 100 000 males in 2009 to 6.6 per 100 000 males in 2017 (https://www3.ha.org.hk/cancereg/facts.html). However, NPC still poses serious socio‐economic and healthcare problems in Hong Kong because the peak incidence of the disease is in the main workforce population with age between 3560 years (≥30 per 100 000 populations in males). Hence, understanding the nature of NPC for the development of effective, target‐specific therapies is still the main research focus in this field.

In NPC, the clonal Epstein‐Barr virus (EBV) genome is consistently detected in both dysplastic lesions and invasive carcinoma, suggesting the crucial role of the virus in cancer progression.^1^, ^2^ EBV resides in NPC as a type II latent infection, in which only latent membrane proteins (LMPs) and EBV nuclear antigen 1 (EBNA1) are expressed.^3^ The oncogenic properties of these viral proteins have been well characterized.^4^, ^5^, ^6^, ^7^, ^8^ Because of their high immunogenic potential, LMPs are usually expressed at low levels in the infected cells to escape the host immune surveillance. In contrast, the non‐immunogenic non‐coding RNAs, such as *EBERs* and viral microRNAs, are abundantly expressed in NPC. EBV was the first virus reported to encode miRNAs, in 2004,^9^ and subsequent work by other teams eventually identified a total of 44 mature EBV‐miRNAs.^10^, ^11^ EBV‐miRNAs are located in two viral genome regions and are named *miR‐BHRF1s* and *miR‐BARTs*. All four *miR‐BHRF1s* are generated in the untranslated region of the early lytic gene, *BHRF1* and are restricted expression in the EBV type III latent infection. However, the rest of the 40 miRNAs derived from the two clusters within the non‐coding *Bam*H1‐A rightward transcripts, *BARTs* (*miR‐BART1* to *miR‐BART22*), are abundantly expressed in all EBV‐positive epithelial malignancies.^12^ The *miR‐BARTs* constitute 38% of the total miRNAs in NPC,^13^ and their diverse functions in augmenting cancer development have been extensively reported; they include maintaining viral latency,^14^, ^15^ promoting survival,^16^, ^17^, ^18^ invasiveness,^19^ metastasis ^20^, ^21^ and controlling the host cells’ immunity.^10^, ^22^, ^23^

Cellular DNA is constantly damaged by different sources of stimuli. Therefore, cells need to preserve genome integrity using the error‐free homologous recombination (HR) pathway for DNA repair. Once DNA double‐strand breaks (DSBs) occur, the ATM rapidly localizes to the damage site and phosphorylates H2AX, which, in turn, recruits a variety of proteins such as BRCA1 and MRE11‐Rad50‐NBS1 (MRN) complexes to form nuclear foci for repairing the damaged DNA. In the nuclear foci, BRCA1‐MRN complexes activate the end resection of DSBs to produce 3’ single‐strand DNA (ssDNA). To ensure accurate DNA correction, RAD52, RPA and RAD51 sequentially bind to the ssDNA and stimulate DNA strand exchange events using the undamaged sister chromatid as a repair template.^24^ Because the availability of sister chromatids is necessary, HR only occurs in the S‐ and G2/M phases of the cell cycle. Both BRCA1 and ATR contribute to HR by activating CHK1 through phosphorylation at S345. The active CHK1 subsequently represses CDK1 activity, resulting in arresting the G2/M checkpoint for cell fate decision; in other words, either HR or apoptosis occurs. Disrupting the activities of HR proteins not only contributes to genomic instability in tumour development but also sensitizes the cells to radio‐ and chemotherapies. We have previously demonstrated that *miR‐BARTs* directly inhibit ATM expression in NPC cells and sensitize the cells to irradiation treatment.^13^, ^25^ Although *BRCA1* mutations have been identified in 54% of NPC in Southeast Europe,^26^ its mutation rate is extremely rare in southern China, where the total *BRCA1/BRCA2/ATM* mutation of 416 NPC cases from four independent genomic studies only accounted for 1.68%.^7^, ^27^, ^28^, ^29^, ^30^ Moreover, weak BRCA1 protein expression is observed in a majority of local NPC cases (57.5%–75%),^31^, ^32^ and promoter hypermethylation has not been detected in NPC cases in either southern China or Southeast Europe.^33^, ^34^ Therefore, BRCA1 expression may be dysregulated by other mechanisms. In fact, several cellular miRNAs, including *miR‐15a‐5p*, *miR‐16‐5p miR‐146a*, *miR‐146b‐5p*, *miR‐182‐5p* and *miR‐638*, have been reported to modulate the cellular DNA damage response (DDR) by directly targeting BRCA1 3’UTR.^35^

In this study, we demonstrated that four *miR‐BARTs* could directly repress BRCA1 expression. More importantly, the suppression of BRCA1 expression by EBV‐miRNAs in the nasopharyngeal epithelial cells diminished the DDR and enhanced the cells’ sensitivity to two common chemotherapeutic agents, cisplatin and doxorubicin.

## 2 MATERIALS AND METHODS

### 2.1 Cell lines, xenografts and patient samples

Four EBV‐positive NPC xenografts (xeno‐666, xeno‐2117, C15 and C17), three EBV‐positive NPC cell lines (C666‐1, NPC43 and C17 cells), two EBV‐negative NPC cell lines (HK1 and NPC53), and an immortalized nasopharyngeal epithelial (NP69), 293FT and HeLa cell lines were used in this study.^13^, ^36^, ^37^, ^38^, ^39^ The clinically frozen specimens for Reverse transcription‐quantitative PCR (RT‐qPCR) (Table S1) and paraffin‐embedded specimens for Immunohistochemistry (IHC) analysis (Table S2) w prospectively collected at the Prince of Wales Hospital, Hong Kong. The EBV status of all NPC cell lines and primary specimens were confirmed by EBER in situ hybridization (ISH) (Figures S1 and S2). The study was approved by the Joint CUHK/NTEC Clinical Research Ethics Committee, Hong Kong.

### 2.2 Reverse transcription‐quantitative PCR

The total RNAs were extracted using the TRIzol reagent (Invitrogen, Carlsbad, CA, USA) and reverse‐transcribed with miScript II RT Kit (Qiagen, Hilden, Germany). The BRCA1 RT‐qPCR product was amplified using the SYBR Green PCR Master Mix Kit (Applied Biosystems, Foster City, CA, USA). The relative gene expressions were normalized with actin, and the fold‐change was calculated using the 2^(∆∆‐^ *^Ct^* ^)^ method. The method for miR‐BART expression has been previously described.^12^ The qPCR primer sequences for BRCA1 and cellular BRCA1‐responsive miRNAs are listed in Table S3.

### 2.3 Immunohistochemistry

The BRCA1‐IHC staining was performed using the Polymer Refined Detection Kit on Leica Bond‐Max, fully automated staining system. The primary antibody for BRCA1 (1:100 dilution, clone MS110; Millipore, Quincy, MA, USA) was used. The expression level of BRCA1 was determined using a scoring system that considered both the staining intensity and prevalence of intensities as described previously.^40^ The specimens with moderate to strong BRCA1 signal were considered IHC positive.

### 2.4 Prediction of microRNA targets

The sequences of miRNAs and the *BRCA1* transcript (NM_007294.2) were extracted from miRBase^41^ and the NCBI, respectively. The putative binding site of *miR‐BARTs* on *BRCA1* was predicted with miRanda and RNAhybrid programs as described previously.^10^, ^42^, ^43^, ^44^ The cut‐off point for the selection was MEF <−16kcal/mol.

### 2.5 The miRNA mimics, inhibitors, expression vectors and transfection

The function of miRNAs was investigated using synthetic, chemically modified, small RNAs that either mimicked (miRNA mimics) or inhibited (miRNA inhibitors) the activity of the specific miRNA in vitro. The BRCA1‐specific siRNAs, miRNA mimics (# B02003) and miRNA inhibitors (# B03001) were synthesized by GenePharm (Shanghai, China). The BRCA1‐siRNAs sequences were as follows: sense, 5’‐GGA AAC CUG UCU CCA CAA AGdTdT‐3’; anti‐sense, 5’‐CUU UGU GGA GAC AGG UUC CdTdT‐3’. The miRNA expression plasmids that contained the miRNA flanking sequence (~300nt), pBART17 and pBART19, were generated by inserting the PCR products into the pcDNA3.1 *via HindIII* and *XhoI* sites. The BRCA1 expression vector, pMH‐SFB‐BRCA1, was obtained from Addgene (plasmid #99394).^45^

In the experiment, 1‐2.5 µg of plasmids, 10 nmol/L of miRNA mimics and 20 nmol/L of siRNAs or miRNA inhibitors were used to transfect the cells in the 6‐well or 12‐well plate format. All transfections were performed using Lipofectamine 2000 (Invitrogen) with standard protocol unless otherwise specified. The stable miR‐BART*‐*expressing HK1 cells were selected with 200 µg/mL of G418 for 6 weeks.

### 2.6 Luciferase reporter assay

The construction of the luciferase reporter plasmids and the procedure of the reporter assay has been previously described.^10^, ^13^ The sequences of oligonucleotides for plasmid construction are listed in the Table S4. The transfection complex containing 200 ng of the reporter vector, 20 ng of the control reporter vector, and 10 nmol/L of individual miRNA mimics were transfected into the 293FT cells in the 24‐well plate format, and the luciferase reporter activities were assayed by Dual Luciferase Reporter Kit after two days.

### 2.7 Drug treatment, IC_50_, cell‐cycle analysis, RAD51 staining, comet assay, clonogenic survival assay

Cisplatin (CDDP) and doxorubicin (DOX) were purchased from LC Laboratories (Woburn, MA) and Pharmachemie BV (Haarlem, The Netherlands), respectively. To determine the IC_50_, the cells were seeded into the 96‐well plate format (2000‐5000 cells/well) overnight, different concentrations of the drugs were added and incubated for another 48 hours. The cell cytotoxicity was assessed using the Cell Counting Kit‐8 (CCK‐8) assay (Dojindo, Kumamoto, Japan) according to the manufacturer's instructions. The IC_50_ was calculated using GraphPad Prism 5 (GraphPad Software Inc, San Diego, CA, USA).

The cell‐cycle analysis, RAD51 staining, comet assays and clonogenic survival assays were described previously with some modifications.^13^, ^46^ In brief, the cells were transfected with the desired siRNAs or miR‐BART mimics for six hours and then evenly seeded into the new cultureware for subsequent analysis. The cells were treated with the specific drug for 24 hours and then stained with propidium iodide for cell‐cycle analysis. For RAD51 staining assays, the cells were seeded on glass coverslips, treated with the drug for 16 hours, returned to growth in the normal medium for three hours, and subsequently fixed and stained with the RAD51 antibody for counting. In the comet assays, the cells were incubated with the specific drug for four hours and then returned to culture for three hours before single‐cell gel electrophoresis was performed using an OxiSelect Comet Assay Kit (Cell Biolabs, San Diego, CA, USA) following the manufacturer's instructions.

For the clonogenic survival assay, 500 or 1000 transfected cells were evenly seeded into the 6‐well plate and exposed with either mock medium or drugs for 24 hours. After 18 days cultured with the growth medium, the colonies were fixed with methanol and then stained with 0.5% crystal violet for visualization. The colonies containing more than 30 cells were counted. The plating efficiency (PE) of the average of the three colonies counted in each treatment was calculated as follows:

$$\text{PE}=\frac{\text{average number of colonies counted from 3 wells}}{\text{number of cells plated}\;}\times 100.$$

Then the surviving fraction was determined with the following equation and compared with the mock treatment control (100%):

$$\text{surviving fraction}=\frac{\text{PE of the treated cells}}{\text{PE of the mock treatment}}\times 100.$$

### 2.8 Antibodies and immunoblotting

The antibodies for BRCA1 (OP92), γ‐H2AX (05‐636) and Vinculin (MAB3574) were purchased from Millipore (Quincy, MA, USA). The antibodies for RAD51 (ab63801), p53 (ab31333) and ATM (ab32420) were purchased from Abcam (Cambridge, MA, USA). Anti‐p21 (#2974), anti‐p‐CHK1 (S345) (#2348) and anti‐PARP1 (#9542) were obtained from Cell Signaling Technology (Danvers, MA, USA). All of the Alexa Fluor‐conjugated and HRP‐conjugated secondary antibodies were purchased from Molecular Probes (New York, NY, USA). Western blot analysis was performed as previously described,^13^, ^47^ and the signal intensity was measured by ImageJ software (http://rsb.info.nih.gov/ij/).

### 2.9 Statistical analysis

Two‐sided Student's *t‐*test was used to compare the differences between the two groups unless otherwise specified. The analysis of each experiment was performed in triplicate, and the results are expressed as mean + SD. All analyses were performed using GraphPad Prism 5.

## 3 RESULTS

### 3.1 Down‐regulation of BRCA1 in NPC

**FIGURE 1:**
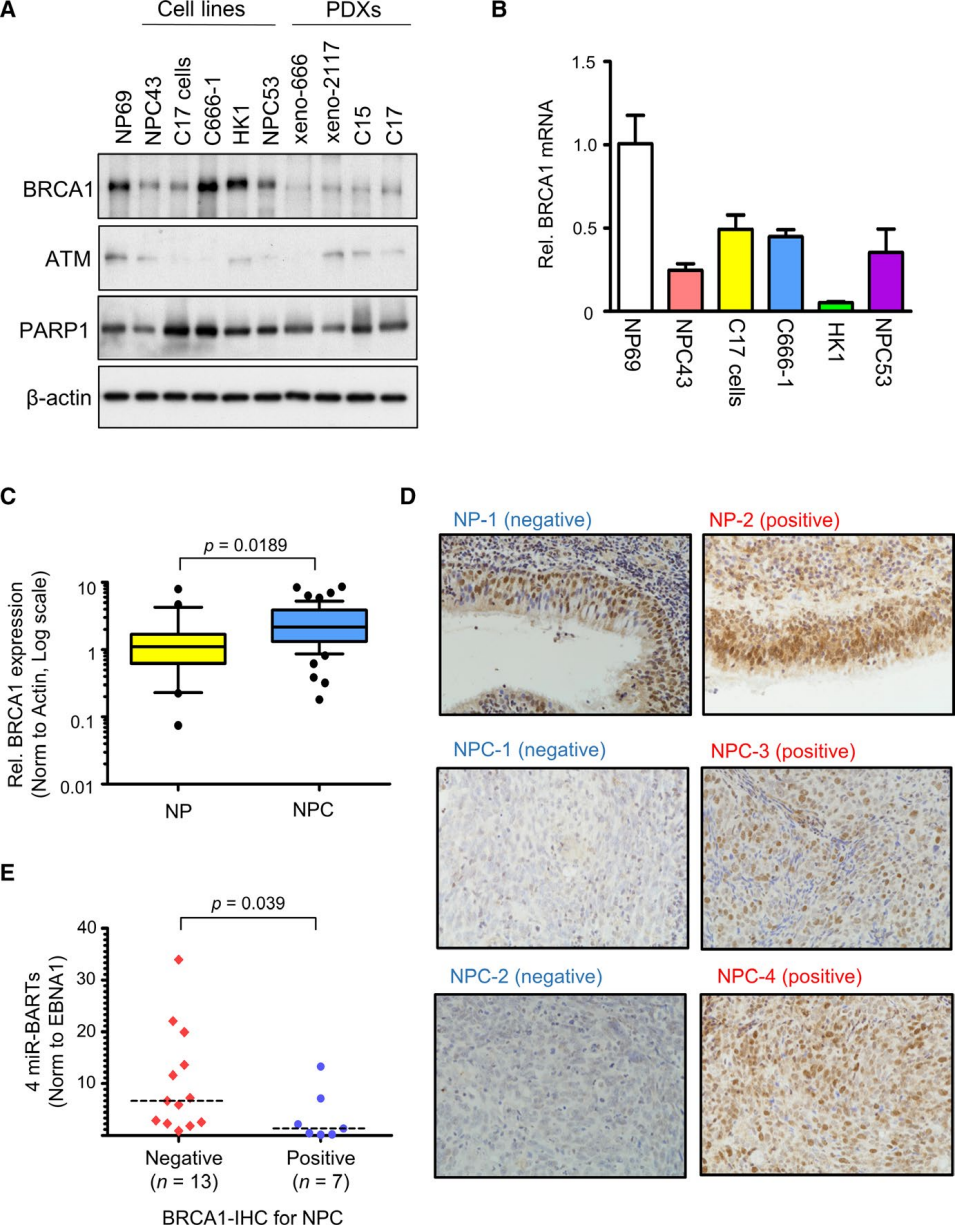
Down‐regulation of BRCA1 in EBV‐associated NPCs. (A) The expression of BRCA1, ATM and PARP1 proteins in immortalized normal NP (NP69), four NPC cell lines and four NPC patient‐derived xenografts (PDXs) were analysed by immunoblotting. Actin was probed as the loading control. (B) The expression levels of BRCA1 mRNA in the cell lines were measured using RT‐qPCR. The relative BRCA1 mRNA expression was calculated using 2(∆∆−Ct) method, and the expression in NP69 was set as 1 for comparison. The data shown is the mean + SD. (C) The whiskers 10‐90 percentiles plot shows the relative BRCA1 mRNA expression in primary samples. The BRCA1 mRNA was significantly up‐regulated in NPCs (n = 55) when compared with the NPs (n = 22). (D) Immunohistochemistry staining of BRCA1 protein in primary samples (number of NPs = 30 and NPCs = 41). The representative images of negative and positive BRCA1 stain in NP and NPC specimens are shown (original magnification X400). (E) The dot plot shows the total expression levels of miR‐BART2‐3p, BART12, BART17‐5p and BART19‐3p in 20 NPC biopsies, in which the BRCA1 protein expression status was analysed in IHC. The expression of miR‐BARTs was normalized to EBNA1. The median values of each group are shown by the dash line and the Mann‐Whitney test was used for the statistical analysis

We have previously reported that several *miR‐BARTs* contribute to the disruption of the DNA damage repair by suppressing the ATM signalling pathway.^13^ Although ATM expression is consistently down‐regulated in NPCs, the expression level of its downstream target, BRCA1, is variable in a panel of NPC samples (Figure 1A). When compared with the immortalized nasopharyngeal epithelial cell line, NP69, the protein levels of BRCA1 in all EBV‐positive NPC patient‐derived xenografts and two EBV‐positive NPC cell lines (NPC43, C17 cells) were highly reduced, whereas high BRCA1 expression was detected in the other three NPC cells, two of which were EBV‐negative (HK1 and NPC53). This observation might indicate that EBV is responsible for the low BRCA1 protein expression in NPC. It is noteworthy that BRCA1 expression in the protein level was not correlated to the mRNA levels among the NPC cell lines (Figure 1B). We subsequently extended our study to examine *BRCA1* expression in primary NPCs. Despite the *BRCA1* mRNA level in NPC tumours (n* = *55) being significantly higher than in non‐cancerous NP tissues (n = 21; *P = *0.0189) in the qRT‐PCR analysis (Figure 1C), reduction of BRCA1 protein expression in NPCs was found in an independent cohort of 30 normal NPs and 41 NPCs (*P* = 0.0005) (Table S5). In our IHC analysis, we detected the predominant positive BRCA1 expression in 83% of NP cases, whereas only 42% of NPC cases scored positive (Figure 1D and Table S5). Since BRCA1 protein expression is not directly correlated to its mRNA level in NPC cell lines and primary tumours, we postulated that BRCA1 expression in NPCs may be regulated in the post‐transcriptional level, likely due to the miRNAs derived from EBV.

### 3.2 BRCA1 is a direct target of *miR‐BARTs*

**FIGURE 2:**
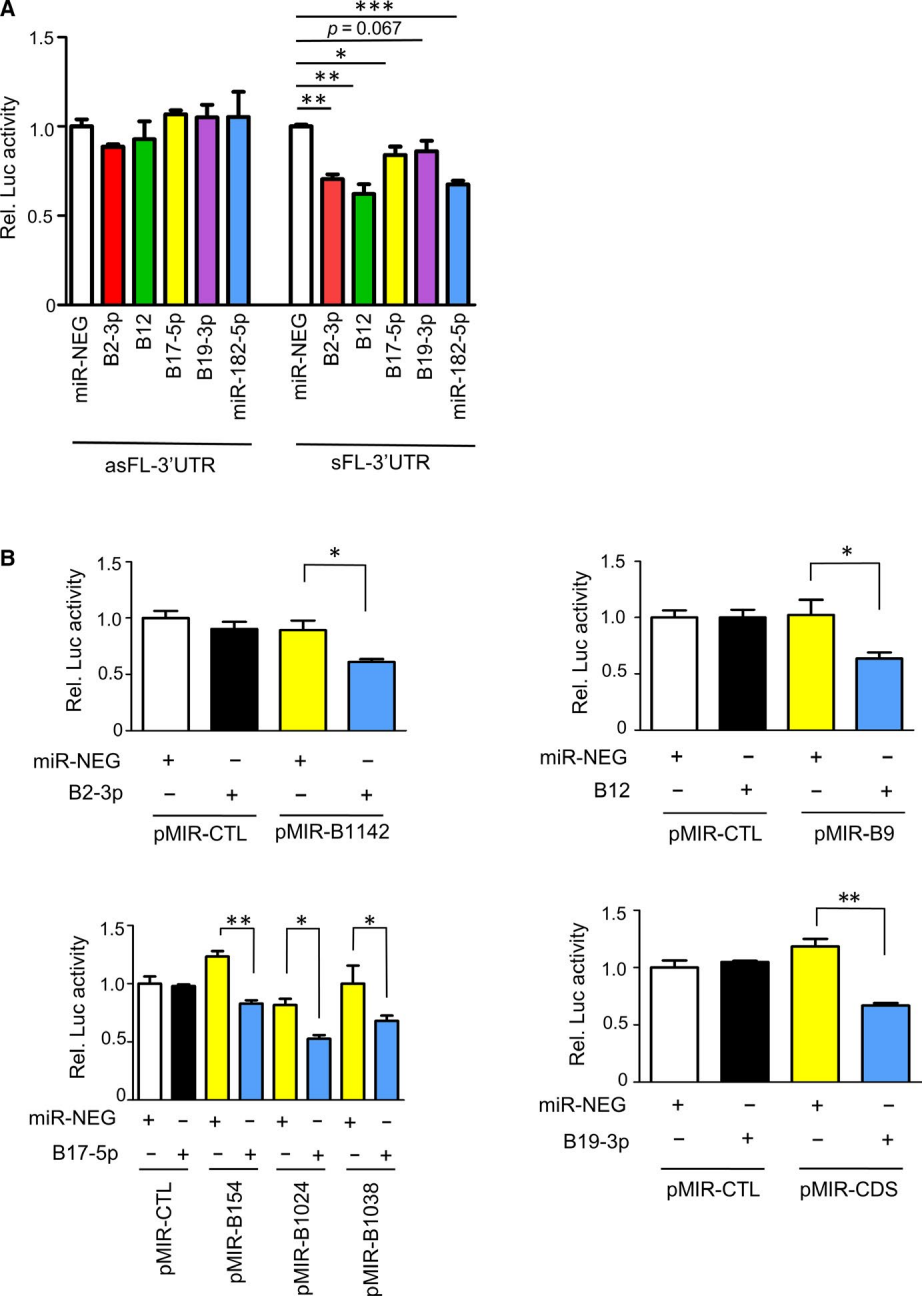
The BRCA1 is the potential target of miR‐BARTs. (A) The relative luciferase activity of the reporter plasmids harbouring a full length of BRCA1‐3’UTR (sFL‐3’UTR) or a full length of BRCA1‐3’UTR in reversed orientation (asFL‐3’UTR) was co‐transfected together with the indicated miRNAs. The luciferase signal with the co‐transfection of negative miRNA mimic control (miR‐NEG) was set at 1 for comparison. (B) The direct interaction between the putative binding sites on BRCA1 and miR‐BARTs were demonstrated in the reporter assays. The firefly luciferase reporter activity was normalized to the Renilla luciferase control. The data shown is the mean + SD from three independent experiments. The result with the co‐transfection of miR‐NEG and pMIR‐CTL was set at 1. pMIR‐CTL = pMIR‐REPORTTM vectors containing unrelated sequences; pMIR‐B = pMIR‐REPORTTM vector harbouring the predicted miR‐BART binding site, pMIR‐CDS = predicted binding site on CDS (Table S5). B2‐3p = BART2‐3p; B12 = BART12; B17‐5p = BART17‐5p; B19‐3p = BART19‐3p. *P < 0.05, **P < 0.001

To further dissect the involvement of EBV in *BRCA1* regulation, we screened the suppressive effect of *miR‐BARTs* on the *BRCA1‐3’UTR* using luciferase reporter assay. As a well‐known BRCA1 modulator, *hsa‐miR‐182‐5p* was included as a positive control.^48^ When compared with the co‐transfection of miRNA control mimics (miR‐NEG), *miR‐182‐5p, BART2‐3p, BART12* and *BART17‐5p* exerted strong suppressive effects on the full length of *BRCA1‐3’UTR*, whereas the inhibitory effects disappeared when the *BRCA1‐3’UTR* was cloned into the reporter in reverse orientation (Figure 2A). Therefore, we predicted the putative binding sites of *miR‐BARTs* by using the default settings of two publicly available computer programs, miRanda and RNAhybrid. In these in silico analyses, a number of putative binding sites of *miR‐BART2‐3p*, *miR‐BART12*, *miR‐BART17‐5p* and *miR‐BART19‐3p* on the *BRCA1* transcript were suggested (Table S6). We subsequently cloned each putative binding site into the 3’UTR of pMIR‐REPORT^TM^ plasmid for luciferase reporter assays. In order to confirm the specificity of the *miRNA* mimics, the 22‐nt of the unrelated sequence was also cloned into the reporter plasmid, pMIR‐CTL, to serve as a negative control. There were no obvious changes in the luciferase signal when the pMIR‐CTL plasmid co‐transfected with any of the tested miRNA or miR‐NEG mimics (Figure 2B). When compared with the transfection of the miR‐NEG in the reporter assays, the repression of luciferase activity was detected in *miR‐182‐5p* positive control (Figure S3). More importantly, luciferase signals were also significantly reduced in six of the 13 predicted binding sites, in which a *BART2‐3p, BART12, BART17‐5p* or *BART19‐3p* mimic and the corresponding reporter plasmids were co‐transfected into 293FT cells (Figure 2B). However, the inhibitory effect was cancelled when the complementarities of the seed region on the binding site was mutated (Figure S4). In line with the reporter assay where *BART19‐3p* weakly interacted with the full length of *BRCA1*‐3’UTR, the only validated putative binding site of *BART19‐3p* was located on the coding sequence (CDS) of *BRCA1*. Taken together, these results suggest that *BART‐2‐3p, BART‐12, BART17‐5p* and *BART19‐3p* can interact with their binding sites on the *BRCA1* transcript to repress protein expression.

### 3.3 Regulation of endogenous BRCA1 expression by miRNAs

**FIGURE 3:**
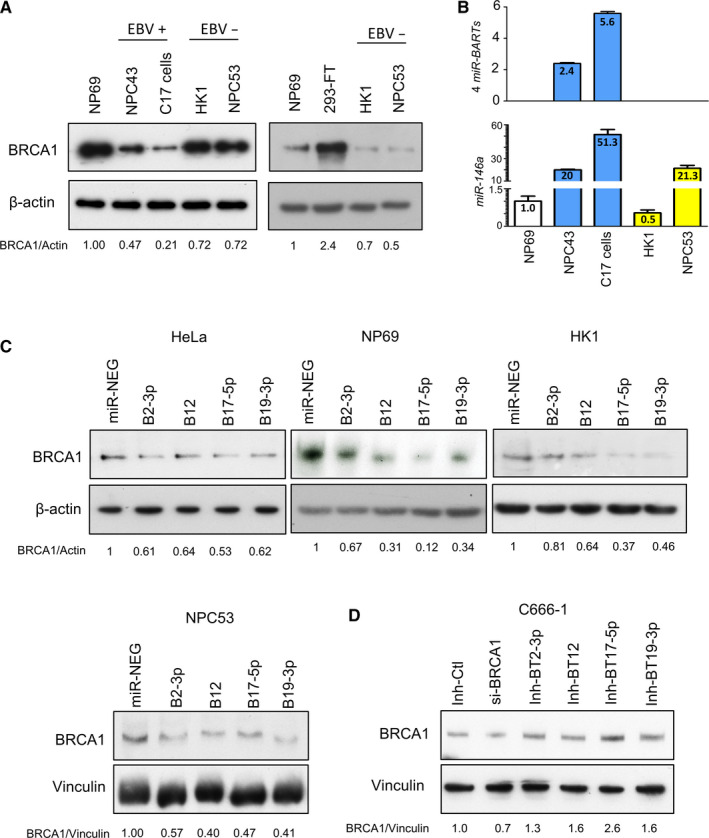
Regulation of BRCA1 expression by miR‐BARTs (A) Western blot of BRCA1 in NPC cell lines. Actin was probed as the protein‐loading control, and the expression level was compared with NP69 (set as 1). (B) The total expression of viral BART2‐3p, BART12, BART17‐5p and BART19‐3p (upper panel) and the expression of cellular miR‐146a (lower panel) in the cell lines were assayed by RT‐qPCR. The expression values of total miR‐BARTs and miR‐146a were calculated using the 2(‐∆Ct) and 2(∆∆‐Ct) methods, respectively. The analysis of each sample was performed in triplicate with mean + SD shown. (C) In the EBV‐negative epithelial cells, the BRCA1 level was suppressed by the transfection of the indicated miRNA mimics, BART2‐3p (B2‐3p), BART12 (BT12), BART17‐5p (BT17‐5p) and BART19‐3p (BT19‐3p). (D) The BRCA1 protein expression in C666‐1 cells was regained by suppressing the endogenous miR‐BARTs activities with specific miR‐BART inhibitors for 48 h. The negative control mimic/inhibitor (Inh‐Ctl) transfection was used for comparison. Either actin or vinculin was probed as the loading control

To dissect the *miR‐BARTs* expression levels in the 20 available NPC biopsies, in which the BRCA1 protein expression status was studied in IHC, we revealed that the total expressions of *BART2‐3p*, *BART12*, *BART17‐5p* and *BART19‐3p* in BRCA1‐positive NPCs (n* = *7) were significantly lower than in BRCA1‐negative NPCs (n = 13) (*P* = 0.039) (Figure 1E). In the cell lines study, the normal nasopharyngeal epithelial cells (NP69) had higher BRCA1 expression than the NPC cell lines in the immunoblotting analysis. Among the NPC cells, the two newly derived EBV‐positive cells (NPC43 and C17 cells) clearly had lower BRCA1 protein levels than the EBV‐negative cells (HK1 and NPC53) (Figure 3A). It is noteworthy that the BRCA1 protein was only barely detected in C17 cells, even though they had similar *BRCA1* mRNA levels to NPC43 (Figure 1B). As the expression levels of most previously reported *BRCA1‐*repressive miRNAs, except *miR‐146a‐5p*, were only slightly different (<4 folds) in NPC cell lines (Figure S5), the relatively high expression levels of total *BART2‐3p, BART12, BART17‐5p* and *BART19‐3p* in C17 cells may be heavily involved in modulating BRCA1 expression in the post‐transcriptional level. The high *miR‐146a‐5p* level in C17 cells may also contribute to the BRCA1 suppression (Figure 3B). The significant difference in *miR‐146a‐5p* expression between HK1 and NPC53 may also result in similar BRCA1 protein expression but considerably different BRCA1 transcript levels between these two EBV‐negative cells (Figure 3A and B).

To directly prove the regulatory effect of *miR‐BARTs* on BRCA1 expression, we introduced the *miR‐BART* mimics into HeLa, NP69, HK1 and NPC53 for analysis. The endogenous BRCA1 protein levels in these four EBV‐negative epithelial cells decreased after the transfection of individual *BART2‐3p, BART12, BART17‐5p and BART19‐3p* mimics for 24 hours (Figure 3C). Moreover, BRCA1 was readily suppressed in the HK1 cells that had been stably transfected with either the BART17 or BART19 expression vector (Figure S6). On the contrary, BRCA1 expression in EBV‐positive C666‐1 cells was increased by suppressing the endogenous *miR‐BARTs* activity with specific miRNA inhibitors (Figure 3D). Overall, the findings provide convincing evidence to support the regulatory role of *BART2‐3p, BART12, BART17‐5p and BART19‐3p* on BRCA1 expression.

### 3.4 Down‐regulation of BRCA1 in NP69 and HK1 cells sensitizes the cells to chemo‐drug treatment

We had previously established a comprehensive transcriptional profile of *miR‐BARTs* in NPC PDXs and realized that *BART2‐3p* (<0.05% of total *miR‐BARTs*) and *BART12* (~0.3%) were expressed at extremely low levels in NPC. In contrast, both *BART17‐5p* (2.5%) and *BART19‐3p* (4.9%) were highly expressed.^13^ Furthermore, these two *miR‐BARTs* exerted a strong BRCA1 repressive effect on transient transfection assays. Hence, we focused on *BART17‐5p* and *BART19‐3p* for the downstream analysis.

**FIGURE 4:**
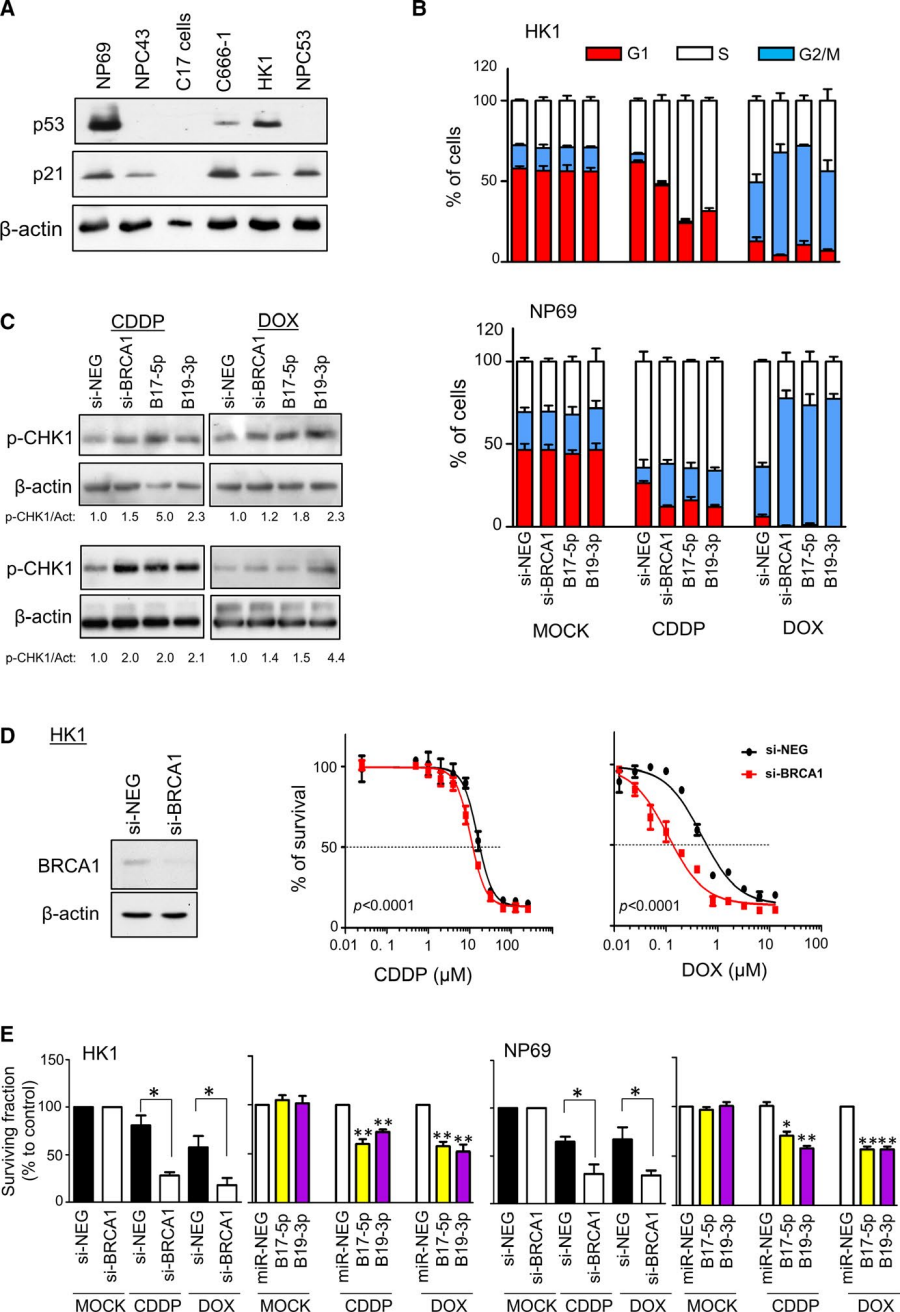
The CDDP and DOX sensitivity in HK1 and NP69 cells. (A) Western blot of p53 and p21 in NPC cell lines were analysed. (B) Transfection of either BRCA1‐specific siRNA, BART17‐5p or BART19‐3p mimics increased CDDP‐ and DOX‐mediated S phase or G2/M phase cell‐cycle arrest in the HK1 and NP69 cells. The transfected cells were incubated with either the control buffer or the indicated chemotherapeutic agent for 24 h. Subsequently, the cells were fixed for DNA content analysis with BD FACSCalibur flow cytometry system. (C) Protein lysate from the treated cells were harvested for phosphor‐CHK1 (p‐CHK1) expression analysis. (D) The suppression of BRCA1 sensitized HK1 cells to CDDP and DOX treatment. HK1 cells were transfected with BRCA1‐specific siRNAs (si‐BRCA1) or siRNA control (si‐NEG) and the protein lysates were collected for BRCA1 expression analysis 24 h after transfection (left panel). The transfected HK1 cells were incubated with different concentrations of CDDP or DOX for 48 h before CCK‐8 analysis. The IC50 value was determined by fitting a sigmoidal dose‐response curve to the data using GraphPad Prism 5 program. Sum‐of‐squares F‐test was used as the comparison method (right panel). (E) Clonogenic survival assays. Approximately 500 or 1000 transfected cells were seeded into the 6‐well plate and treated with CDDP or DOX for 24 h. The cells were cultured for 14‐18 d in normal medium before staining, and colonies containing more than 30 cells were counted. The number of colonies generated from the mock treatment was compared (set as 100%). All the experiments were performed in triplicate and the Student's *t*‐test was conducted, compared with the control transfected cells. *P < 0.05; **P < 0.01

Since BRCA1 is a critical component in the DDR pathway, the *miR‐BARTs‐*mediated down‐regulation of BRCA1 in NPC is hypothesized to increase the cell sensitivity to chemotherapeutic agents. To test this hypothesis, we determined the IC_50_ of cisplatin (CDDP) and doxorubicin (DOX) in the available NPC cells. The IC_50_ values of CDDP and DOX were varied among the cell lines, but the EBV status had no correlation to drug sensitivity (Table 1). It was previously reported that p53 is highly expressed in over 90% of NPCs,^49^ but we only detected p53 expression in NP69, C666‐1 and HK1 cells (Figure 4A). As p53 plays a critical role in eliciting the DDR to cell‐cycle control and apoptosis, we attempted to prove the importance of *miR‐BARTs* in response to CDDP and DOX treatments by suppressing BRCA1 expression in the p53‐expressing cells, NP69 and HK1, with siRNAs or *miR‐BART* mimics.

**TABLE 1:** The cell sensitivity to the DNA‐damaging chemotherapeutic drugs

Cell line	EBV status	P53 protein	Cisplatin (µmol/L)				Doxorubicin (µmol/L)		Olaparib^a^ (µmol/L)
			IC_30_	IC_50_	IC_70_	IC_30_	IC_50_	IC_70_	IC_50_
NP69	−	+	4.00	5.20	6.75	0.33	0.44	0.59	Insensitive
C666‐1	+	+	14.34	15.97	29.40	1.09	1.24	1.41	Insensitive
NPC43	+	−	10.57	13.51	17.28	1.54	4.28	11.87	Insensitive
C17 cell	+	−	2.20	4.43	9.25	0.08	0.17	0.36	Insensitive
HK1	−	+	9.40	15.59	16.70	0.44	1.02	2.40	Insensitive
NPC53	−	−	1.80	3.62	7.20	0.70	0.86	0.93	Insensitive
SW620^b^									54.64
A549									31.31
HeLa									90.40

^a^ Maximum 300 µmol/L of Olaparib was tested.

^b^ SW620 is a colon cancer cells with no BRCA1 gene mutations or DSBR defects and suggest to be highly resistant to Olaparib.

We examined whether the suppression of BRCA1 could potentiate the cytotoxic effect of CDDP and DOX with flow cytometry analysis. When compared with the control transfected cells, HK1 and NP69 transfected with a si‐BRCA1, *BART17‐5p* or *BART19‐3p* mimic remarkably induced cell‐cycle arrest at S phase or G_2_/M phase after exposure to CDDP (7.5 µmol/L for HK1 and 2 µmol/L for NP69) and DOX (0.25 µmol/L for HK1 and 0.1 µmol/L for NP69) for 24 hours (Figure 4B). Consistent with the cell‐cycle analysis, the elevation of the phosphor‐Chk1 level was detected in the BRCA1 knockdown cells after treatments (Figure 4C). As a control, the transfected cells alone did not affect the normal cell‐cycle progression (Figure 4B and Figure S7). This data suggests that both CDDP‐ and DOX‐induced DNA damage in the BRCA1‐deficient cells may activate the ATR‐CHK1 pathway to trigger G2/M arrest to block cell proliferation and induce apoptosis. In agreement with cell‐cycle findings, the transfection of BRCA1‐specific siRNA into HK1 cells notably sensitized the cells to CDDP (si‐NEG: IC_50_ = 15.59 µmol/L; si‐BRCA1: IC_50_ = 10.59 µmol/L, *P < *0.0001) and DOX (si‐NEG: IC_50_ = 0.48 µmol/L; si‐BRCA1: IC_50_ = 0.11 µM, *P < *0.0001) after 48 hours of incubation (Figure 4D). In contrast, no improvement of the drug efficacy was detected when the si‐BRCA1 was transfected into NP69 or when *miR‐BART17‐5p* and *miR‐BART19‐3p* mimics were transfected into HK1 and NP69 cells (data not shown). In the short‐term experiment, the drug cytotoxicity on the *miR‐BART* transfected cells may be diminished because *miR‐BART17‐5p* and *miR‐BART19‐3p* can also promote cell proliferation via *NF‐κB* signalling and Wnt signalling pathways, respectively.^50^, ^51^ Although discordant findings in IC_50_ comparison were observed, the long‐term clonogenic survival study further confirmed the enhancement of CDDP‐ and DOX‐induced cytotoxic effects by *miR‐BARTs* in both HK1 and NP69. When compared with the control, the si‐BRCA1 transfected HK1 and NP69 cells remarkably reduced the colony formation rates by more than 50% after incubation with CDDP (7.5 µmol/L for HK1 and 2 µmol/L for NP69) or DOX (0.25 µmol/L for HK1 and 0.1 µmol/L for NP69) for 24 hours. Similarly, the increased *BART17‐5p* or *BART19‐3p* level in the cells suppressed colony formation, although the magnitude of the effect was clearly reduced (Figure 4E and Figure S8).

**FIGURE 5:**
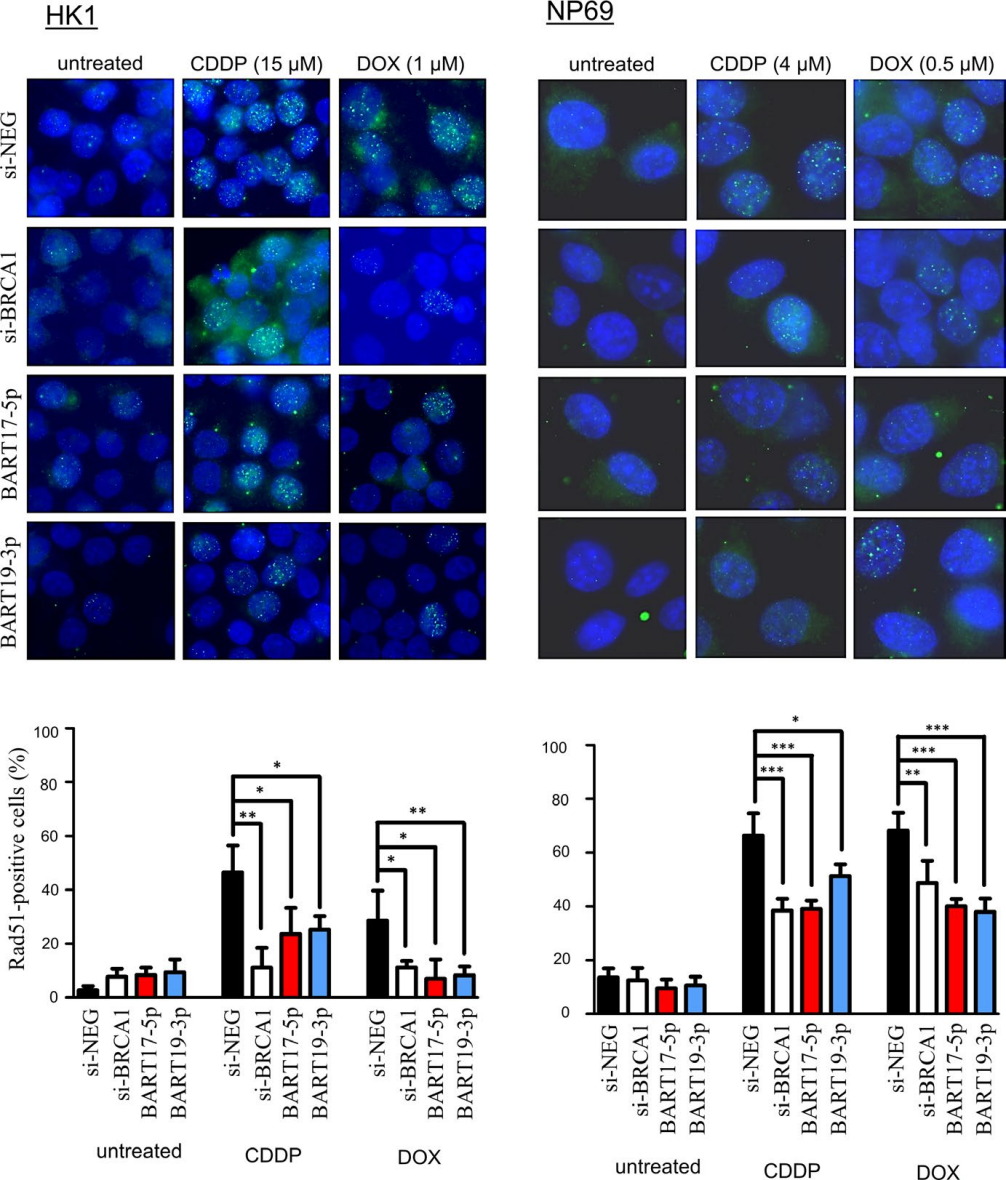
The EBV‐miRNAs impair cisplatin‐ and doxorubicin‐induced DNA damage response in nasopharyngeal epithelial cells. The representative images of the RAD51 foci staining in HK1 cells (upper left panel) and NP69 cells (upper right panel) are shown. The cells transfected with either siRNA control (si‐NEG), BRCA1‐specific siRNA (si‐BRCA1) or miR‐BARTs mimics were treated with cisplatin (CDDP) and doxorubicin (DOX), followed by immunostaining with the RAD51 antibody. At least 100 nuclei were randomly selected for counting, and the cells containing more than five apparent RAD51 foci in the nucleus were considered positive. The percentage of the RAD51‐positive cells with mean + SD from three independent experiments are shown in the lower panel. Student's *t*‐test was used to compare them with the control transfected cells (miR‐NEG) in each set of experiments. *P < 0.05; **P < 0.01; ***P < 0.001

**FIGURE 6:**
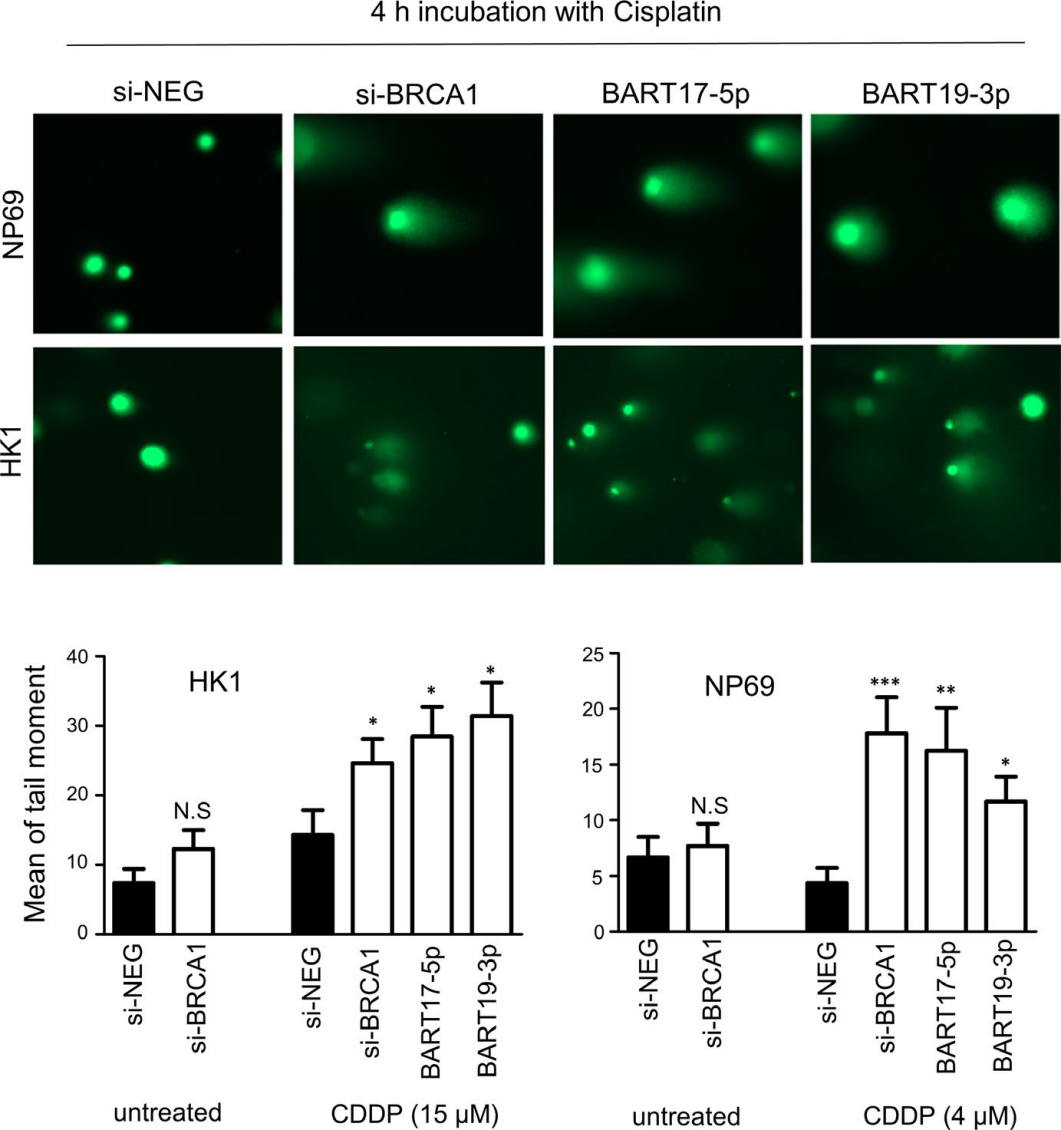
The overexpression of miR‐BARTs increase DNA damage response in nasopharyngeal epithelial cells. The transfected HK1 and NP69 cells were treated with the indicated concentration of cisplatin (CDDP) for four hours and subsequently returned to the fresh culture medium for three hours before they were analysed by comet assays. The representative images for different transfected cells are shown in the upper panel. At least 70 cells were randomly selected for analysis using ImageJ software. The mean of the tail moment is shown in the bar chart: mean + SEM. Student's *t*‐test was used to compare them with the miR‐NEG transfected control (black bar). *P < 0.05; **P < 0.01; ***P < 0.001

To investigate whether the increased cytotoxic effect of the transfected cells was caused by defects in the DNA repair system, we extended our study to examine the DNA damage recovery ability of the cells with a RAD51 foci formation assay (Figure 5). Since RAD51 bound to the DNA damage site to initiate HR, the number of RAD51 foci in the cells was directly correlated to the efficiency of the DNA damage repair. In the absence of drug treatment, the number of positive RAD51 nuclear foci in the BRCA1 knockdown and *miR‐BART* expressing cells were in the background level, with no apparent difference to the control transfected cells. As expected, incubating the control transfected HK1 and NP69 cells in CDDP (HK1 = 15 µmol/L; NP69 = 4 µmol/L) or DOX (HK1 = 1 µmol/L; NP69 = 0.5 µmol/L) for 16 hours sharply induced RAD51 foci formation. The formation of RAD51 foci in the *miR‐BART*‐expressing cells increased as well. However, the percentage of cells with RAD51‐positive foci was significantly reduced in both *miR‐BART* and si‐BRCA1 transfected cells when compared with the control (*P* < 0.05), suggesting that the efficiency of the DNA repair system was attenuated by either suppressing BRCA1 function or increasing *miR‐BARTs* activity. In compliance with the RAD51 nuclear foci staining results, our comet assays further indicated that all *miR‐BART‐*expressing cells and BRCA1 knockdown cells had higher DNA damage levels with the same concentration of CDDP for four hours (Figure 6). Taken together, our findings provide strong evidence that the abundantly expressed EBV‐encoded miRNAs, *BART17‐5p* and *BART19‐3p,* can promote the cisplatin and doxorubicin sensitivity of the NPCs, likely due to the suppression of BRCA1 activity.

## 4 DISCUSSION

The DNA damage response (DDR) is a complex signalling network that attenuates the DNA damage and maintains genomic stability in the cells. Impairing the DDR may contribute to the initiation of tumour development and make the cells more sensitive to DNA‐damaging agents. In our recent whole‐genome sequencing study on 70 NPCs, we uncovered that NPC patients with somatic defects in DDR genes (TP53, ATM, BRCA1 and BRCA2) had significantly shorter survival rates than those with null mutation (KWL, unpublished observation). Several research teams have recently demonstrated the important roles of miRNAs in regulating the expression of specific DNA repair factors.^52^

In this study, we identified that BRCA1, a tumour suppressor gene responsible for DNA double‐strand break‐repair, is the cellular target of *BART2‐5p, BART12, BART17‐5p* and *BART19‐3p,* in which *BART17‐5p* and *BART19‐3p* constitute ~8.6% of the total viral miRNAs in NPC.^13^ The direct interaction of *miR‐BARTs* on six in silico predicted binding sites on the BRCA1 3’UTR was confirmed using reporter assays (Figure 2B). Interestingly, the miRNA response element (MRE) of *BART19‐3p* is located on the CDS of *BRCA1*. Although miRNAs commonly suppress target gene translation by binding with their 3’UTRs, their MRE can mechanistically occur in any position on the target mRNA.^53^ Moreover, the MRE located on the CDS can be validated by luciferase assays, with a reporter vector containing the specific MRE in the 3’UTR.^54^ In line with our findings, one of the *BART17‐5p* (BRCA1‐1038) and *BART19‐3p* (BRCA1‐CDS‐2694) binding sites were previously identified in the primary effusion lymphoma cell lines using the highly sensitive PAR‐CLIP method.^55^ We further found that the inhibition of BRCA1 or overexpression of *BART17‐5p* and *BART19‐3p* in HK1 and NP69 cells can make the cells more susceptible to cisplatin and doxorubicin treatments (Figure 4D and E).

This study had limitations as well. We have demonstrated that overexpressing *miR‐BARTs* in EBV‐negative cells leads to increased cell sensitivity to chemotherapeutic drugs. However, we do not have suitable EBV‐positive NPC cell lines to prove the hypothesis in the miR‐BART knockdown experiment. There are only three EBV‐positive NPC cell lines available worldwide, but both NPC43 and C17 cells were established from recurrent NPC^37^, ^39^ and have characteristics different from those of primary NPC; for instance, they have no p53 protein expression (Figure 4A). The C666‐1 is the model commonly used for the study of viral‐host interaction, and the positive correlation between endogenous BRCA1 expression and the chemoresistance of the drugs can be demonstrated in C666‐1 (Figure S9). However, both BRCA1 protein and *miR‐BARTs* were highly expressed in C666‐1, indicating that other mechanisms in C666‐1 may diminish the involvement of *miR‐BARTs* in BRCA1 regulation, such as shortening the BRCA1 3’UTR.^56^

Together with our previous findings, we have identified the roles of eight *miR‐BARTs* in regulating two essential DNA homologous recombination factors, ATM and BRCA1. These eight miRNAs occupy about 20% of the total EBV‐encoded miRNAs in NPCs.^13^ The repression of either ATM or BRCA1 expression makes the cells hypersensitive to the treatment with olaparib, an FDA approved PARP inhibitor.^48^, ^57^, ^58^ Hence, the relationship between the effect of olaparib, BRCA1 expression and *miR‐BARTs* activity should be evaluated in NPC. Unfortunately, the NPC cell lines available in our laboratory were highly resistant to olaparib (concentration ≤ 300 µmol/L), albeit PARP1 proteins were abundantly expressed (Table 1 and Figure 1A). Thus, the effect of olaparib on NPC was excluded from the study.

This study is the first report to demonstrate the interaction of four *miR‐BARTs* on the *BRCA1* transcript. Our findings at least partially support the hypothesis that DNA repair factors are tightly regulated by *miR‐BARTs* during tumorigenesis in a group of EBV‐infected NPCs. Since overexpression of *miR‐BARTs* can potentiate the sensitivity to chemotherapeutic agents in some nasopharyngeal epithelial cells, our study may contribute to the development of effective therapies for NPC management.

## CONFLICT OF INTEREST

The authors confirm that there are no conflicts of interest.

## AUTHOR CONTRIBUTION

**Raymond Wai‐Ming Lung:** Conceptualization (supporting); Data curation (lead); Formal analysis (lead); Investigation (supporting); Supervision (supporting); Writing‐original draft (lead); Writing‐review & editing (supporting). **Joanna Hung‐Man Tong:** Conceptualization (supporting); Data curation (supporting); Formal analysis (lead); Investigation (supporting); Methodology (lead); Supervision (lead). **Lok‐Man Ip:** Data curation (supporting); Formal analysis (supporting); Methodology (supporting). **Ka‐Hei Lam:** Data curation (supporting); Formal analysis (supporting). **Anthony Wing‐Hung Chan:** Formal analysis (lead); Methodology (lead). **Wing‐Po Chak:** Data curation (supporting); Formal analysis (supporting). **Lau‐Ying Chung:** Data curation (supporting). **Walter Wai Yeung:** Data curation (supporting); Formal analysis (supporting); Methodology (supporting). **Pok‐Man Hau:** Conceptualization (supporting); Investigation (supporting). **Shuk‐Ling Chau:** Data curation (supporting); Methodology (supporting). **Sai Wah Tsao:** Investigation (supporting); Resources (lead). **kin‐Mang Lau:** Conceptualization (supporting); Funding acquisition (supporting); Investigation (supporting); Writing‐original draft (supporting). **Kwok‐Wai Lo:** Conceptualization (lead); Funding acquisition (supporting); Investigation (supporting); Resources (lead); Supervision (lead); Writing‐original draft (supporting); Writing‐review & editing (supporting). **Ka Fai To:** Conceptualization (lead); Formal analysis (lead); Funding acquisition (lead); Investigation (lead); Project administration (lead); Resources (lead); Supervision (lead); Writing‐original draft (lead).

## Supporting information

Appendix S1

## Data Availability

The data that supports the findings of this study are available in the Appendix S1 of this article.
