# Supplementary material for: EBV–encoded miRNAs can sensitize nasopharyngeal carcinoma to chemotherapeutic drugs by targeting BRCA1
Source: J Cell Mol Med. 2020 Oct 19;24(22):13523–35. doi: 10.1111/jcmm.16007 (PMC7701581; doi:10.1111/jcmm.16007)
Supplement: Supplementary file 1 — Appendix S1 [file JCMM-24-13523-s001.docx]

***Supporting Information***

**EBV–encoded miRNAs sensitize nasopharyngeal carcinoma to chemotherapy drugs by targeting BRCA1**

Running title: Regulation of BRCA1 by EBV-miRNAs in NPC

Raymond Wai-Ming Lung^1^, Joanna Hung-Man Tong^1^, Lok-Man Ip^1^, Ka-Hei Lam^1^, Anthony Wing-Hung Chan^1^, Wing-Po Chak^1^, Lau-Ying Chung^1^, Walter Wai Yeung^1^, Pok-Man Hau^1^, Shuk-Ling Chau ^1^, Sai-Wah Tsao^3^, Kin-Mang Lau^1^, Kwok-Wai Lo^1^, Ka-Fai To^1,2, *^

Affiliations:

^1^ Department of Anatomical and Cellular Pathology, State Key Laboratory in Oncology in South China. Prince of Wales Hospital, The Chinese University of Hong Kong, HKSAR, China

^2^ Institute of Digestive Disease, Partner State Key Laboratory of Digestive Disease, The Chinese University of Hong Kong, HKSAR, China

^3^ Department of Biomedical Sciences, The University of Hong Kong, Pokfulam, Hong Kong.

^#^ *Corresponding Author*:

**Ka-Fai To:**

Department of Anatomical and Cellular Pathology, Prince of Wales Hospital, The Chinese University of Hong Kong, Shatin, N.T. Hong Kong

Phone: 852-3505-3337

Fax: 852-2637-6274

**Table S1.** Characteristics of the primary specimens recruited for quantitative RT-qPCR analysis

| **Frozen specimens for RT-qPCR analysis** | | | | |  |
| --- | --- | --- | --- | --- | --- |
| **Variables** | **NP** | **%** | **NPC** | **%** | ****P* value** |
| **Total No of patients** | 22 |  | 53 |  |  |
| **Age (years)** |  |  |  |  | 0.4412 |
| ≤ 50 | 15 | 68 | 30 | 57 |  |
| > 50 | 7 | 32 | 23 | 43 |  |
| Mean | 46.45 |  | 48.84 |  |  |
| **Gender** |  |  |  |  | 0.1573 |
| Male | 13 | 60 | 41 | 77 |  |
| Female | 9 | 40 | 12 | 23 |  |
| **Clinical stage** |  |  |  |  |  |
| Early (stages 1 and 2) | N.A. |  | 27 | 51 |  |
| Late (stages 3 and 4) | N.A. |  | 20 | 38 |  |
| Information not available | N.A. |  | 6 | 11 |  |

N.A.: not applicable.

**P* value was analyzed using Fisher’s exact test.

**Table S2.** Characteristics of the primary specimens recruited for IHC analysis

| **Formalin-fixed paraffin-embedded specimens for IHC analysis** | | | | |  |
| --- | --- | --- | --- | --- | --- |
|  | NP | % | NPC | **%** | ***P* value*** |
| **Total No of patients** | 30 |  | 41 |  |  |
| **Age (years)** |  |  |  |  | 0.4666 |
| ≤ 50 | 17 | 57 | 27 | 66 |  |
| > 50 | 13 | 43 | 14 | 34 |  |
| Mean | 50.53 |  | 48.48 |  |  |
| **Gender** |  |  |  |  | 0.7797 |
| Male | 22 | 73 | 32 | 78 |  |
| Female | 8 | 27 | 9 | 22 |  |
| **Clinical stage** |  |  |  |  |  |
| Early (stages 1 and 2) | N.A. |  | 19 | 46 |  |
| Late (stages 3 and 4) | N.A. |  | 12 | 29 |  |
| Information not available | N.A. |  | 10 | 25 |  |

N.A.: not applicable.

**P* value was analyzed using Fisher’s exact test.

**Table S3:** The sequences of oligonucleotides used for quantitative RT-PCR analysis

| Primer Name | Sequence (5’ to 3’) |
| --- | --- |
| BRCA1 (forward) | GAA ACC GTG CCA AAA GAC TTC |
| BRCA1 (reverse) | CCA AGG TTA GAG AGT TGG ACA C |
| *QRT-BART2-3p | AGG AGC GAT TTG GAG AAA ATA A |
| *QRT-BART12 | CTG TGG TGT TTG GTG TGG TT |
| *QRT-BART17-5p | TGTATGCCTGGTGTCCCCTTAGT |
| *QRT-BART19-3p | TTTTGTTTGCTTGGGAATGCT |
| *miR-15a-5p | TAG CAG CAC ATA ATG GTT TGT |
| *miR-16-5p | TAG CAG CAC GTA AAT ATT GGC |
| *miR-146a-5p | TGA GAA CTG AAT TCC ATG GG |
| *miR-146b-5p | TGA GAA CTG AAT TCC ATA GGC |
| *miR-182-5p | TTT GGC AAT GGT AGA ACT CAC |
| *miR-187-5p | GGC TAC AAC ACA GGA CCC GGG C |
| *miR-638 | AGG GAT CGC GGG CGG GTG GC |
| *U6 | ACG CAA ATT CGT GAA GCG TT |
| β-actin (forward) | CTG GCA CCC AGC ACA ATG |
| β-actin (reverse) | GCC GAT CCA CAC GGA GTA CT |

*The primer was used in the miScript SYBR Green PCR kit.

**Table S4.** The sequences of oligonucleotides for luciferase reporter cloning

| **Name** | **Sequence (5' to 3')** | **Remark** |
| --- | --- | --- |
| **BRCA1-3’UTR -F** | AGA CAC TAG TAC TGC AGC CAG CCAC | For full length 3’UTR PCR cloning |
| **BRCA1-3’UTR –R** | GAG AAG CTT ACC TAG TCC TTC CAA CAG CT |  |
| **BRCA1 reversed-3’UTR- F** | AGA CAC TAG TAC CTA GTC CTT CCA ACA GCT | For full length 3’UTR PRC cloning (reverse orientation) |
| **BRCA1 reversed-3’UTR-R** | GAG AAG CTT ACT GCA GCC AGC CAC |  |
| **miR-148-5p-S** | CTAGTGCCTGAGTGTATAACAGAACTTA | For pMIR-CTL |
| **miR-148-5p-AS** | AGCTTAAGTTCTGTTATACACTCAGGCA |  |
| **BRCA1-1142-S** | CTA GTG ATT TTT TTC CTT TGC TCC CTG A | *miR-BART2—3p* |
| **BRCA1-1142-AS** | AGC TTC AGG GAG CAA AGG AAA AAA ATC AA |  |
| **BRCA1-9-S** | CTA GTA GCC ACA GGT ACA GAG CCA CAG GAC A | *miR-BART12* |
| **BRCA1-9-AS** | AGC TTG TCC TGT GGC TCT GTA CCT GTG GCT A |  |
| **BRCA1-154-S** | CTA GTT GGC TAT GCA AGG GTC CCT TAA A | *miR-BART17—5p* |
| **BRCA1-154-AS** | AGC TTT TAA GGG ACC CTT GCA TAG CCA A |  |
| **BRCA1-1024-S** | CTA GTG TTG TAG CTC TGG TAT ATA ATC CAT TCC TCT TAA A | *miR-BART17—5p* |
| **BRCA1-1024-AS** | AGC TTT TAA GAG GAA TGG ATT ATA TAC CAG AGC TAC CAC A |  |
| **BRCA1-1038-S** | CTA GTA TAT AAT CCA TTC CAC TTA A | *miR-BART17—5p* |
| **BRCA1-1038-AS** | AGC TTT AAG AGG AAT GGA TTA TAT A |  |
| **BRCA1-2694-S** | CTA GTA GTC ACT TTT GAA TGT GAA CAA AAA | *miR-BART19—3p* |
| **BRCA1-2694-AS** | AGC TTT TTT GTT CAC ATT CAA AAG TGA CTA |  |
| **BRCA1-874-S** | CTA GTA TGA AAC TAG AAG AGA TTT CTA AAA A | *hsa-miR-182-5p* |
| **BRCA1-874-AS** | AGC TTT TTT AGA AAT CTC TTC TAG TTT CAT A |  |
| **BRCA1-1137-S** | CTA GTG GTG ATT TTT TTC CTT TGC TCC CTG TTG CTG AAA | *hsa-miR-182-5p* |
| **BRCA1-1137-AS** | AGC TTT TCA GCA ACA GGG AGC AAA GGA AAA AAA TCA CCA |  |

**Table S5:** Expression of BRCA1 in primary NP and NPC cases

| **Sample** | **BRCA1** | | **Total** | ***p*-value**  **(Fisher’s exact test)** |
| --- | --- | --- | --- | --- |
|  | **Positive (%)** | **Negative (%)** |  |  |
| **NPC** | 17 (42.5%) | 24 (58.5%) | 41 | 0.0005 |
| **NP** | 25 (83.3%) | 5 (16.7%) | 30 |  |

**Table S6: Predicted *miRNA* binding sites on *BRCA1* transcript**

| *MicroRNA | Transcript location | Alignment | MEF  (kcal/mol) |
| --- | --- | --- | --- |
| BART2-3p | 59-88 (3’UTR) | **BRCA1-59: 5’ GUGGCCUUUCCAGGCCCUGGGAGCUCCUC 3’**  **\| \|:\|\|\|\|:: \| \|\|\|\|\|\|**  **BART2-3p: 3’ AAUAAAAGAGGUUUAG------CGAGGAA 5’** | -21.5 |
| BART2-3p | 174-200  (3’UTR) | **BRCA1-174: 5’ AGAUUUUCUGCUUGAAGUCUC-CCUUG 3’**  **\|\|\|\|\|\|\| \|: \|\|:\|\| \| \|\|\|\|**  **BART2-3p: 3’ AAUAAAAGA-GG--UUUAGCGAGGAA 5’** | -18.7 |
| BART2-3p | 971-997  (3’UTR) | **BRCA1-971: 5’ AACCGGUUUCCAAAGACAGUCUUCUA 3’**  **\|:\|\|\|\|\|\| \| \|\|:\|\|**  **BART2-3p: 3’ AAUAAAAGAGGUUUAG--C-GAGGAA 5’** | -19.0 |
| BART2-3p | 1142-1165  (3’UTR) | **BRCA1-1142: 5’ UGAUUUUUUUCCU--UUGCUCCCUG 3’**  **\|:\| \|\|\|\|:\|\|\| \| \|\|\| \|\|\|**  **BART2-3p: 3’ AAU-AAAAGAGGUUUAGCGA-GGAA 5’** | -24.4 |
| BART12 | 9-34  (3’UTR) | **BRCA1-9: 5’ AGCCACAGGUACAGA-GCCACAGGAC 3’**  **\|:\|\|\|\|\| \|\|:\| :\|\|\|\|\|\|\|\|**  **BART12: 3’ UUGGUGUG---GUUUGUGGUGUCCU 5’** | -31.5 |
| BART12 | 588-619  (3’UTR) | **BRCA1-588: 5’ GGCUCACGCCUGUAAUCCCAGCACUUUGGGAG 3’**  **:\| \|\|\|:\|\| \|:\|\|\|: :\|\|\|**  **BART12: 3’ UUG-GUGUGGU--------UUGUGGUGUCCU 5’** | -26.4 |
| BART17-5p | 154-175  (3’UTR) | **BRCA1-154: 5’ UGGCUAUGCAAGGGUCC-CUUAA 3’**  **\|\|\|\|\| \| \|\|\|\| \|\|\|\|**  **BART17-5p: 3’ GAACAUACGGACGCAGGAGAAU 5** | -23.4 |
| BART17-5p | 1024-1057  (3’UTR) | **BRCA1-1024: 5’ GUUGUA-GCUCUG-GUAUAUAAUCCAUUCCUCUUAA 3’**  **\|\|\|\|\| \|\| \|\|\| \| \|\|\|:\|\|\|\|**  **BART17-5p: 3’ GAACAUACG-GACGC------------AGGAGAAU 5’** | -22.6 |
| **BART17-5p | 154-175  (3’UTR) | **BRCA1-1038: 5’ AUAUAAU-CCAU--UCCUCUUA 3’**  **\| \|\| \|\| \|\|\|\|\|\|\|\|**  **BART17-5p: 3’ GAACAUACGGACGCAGGAGAAU 5’** | -16.0 |
| **BART19-3p | 2694-2717  (CDS) | **BRCA1-CDS-2694: 5’ AGUCACUUUUGAAUGUGAACAAAA 3’**  **\|\| \|\| \|\| \|\| \| \|\|\|\|\|\|\|**  **BART19-3p: 3’ UC-GU-AAGGGUU-CGUUUGUUUU 5’** | -15.9 |
| BART19-3p | 535  (3’UTR) | **BRCA1-535: 5’ AGGAAAUUCUGAGGCAGGUAUUA 3’**  **\|\|: \|\|\|::\| \|\|\| :\| \|**  **BART19-3p: 3’ UCGU--AAGGGUUCGUUUGUUUU 5’** | -19.4 |
| BART19-3p | 733  (3’UTR) | **BRCA1-733: 5’ UGUAAUCCCAGCUACUCAGGUGGCUAAGGCAGGAG 3’**  **\|:\| \|\|\|\|\| :\|\| \|\| \|\|::\|**  **BART19-3p: 3’ UCGUAAGGGU-----------UCGUUU—-GUUUU 5’** | -19.5 |
| BART19-3p | 1278  (3’UTR) | **BRCA1-1278: 5’ AGCCCCCCCAGUGUGCAAGGGCAGUGAAGAC 3’**  **\|\|\| \|\|\|\| :\|\|\|: \|\|:\|**  **BART19-3p: 3’ UCGUAAGGGU---------UCGUUUGUUUU 5’** | -19.5 |
| †miR-182-5p | 874-898  (3’UTR) | **BRCA1-874: 5’ AUGAAACU-AG-AAGAG-AUUUCUAAAA 3’**  **\| \| \| \|\| \| \|\|\| \|\|\|\|\|**  **miR-182-5p: 3’ U-C--ACACUCAAGAUGGUAACGGUUU 5’** | N.A. |
| miR-182-5p | 1137-1169  (3’UTR) | **BRCA1-1137: 5’ GGUGAUUUUUUUCCUUUGCUCCCUGUUGCUGAA 3’**  **\|\|\|\| \|\|: \|:\|: :\|\|\|\|::\|\|**  **miR-182-5p: 3’CACACUCAAG-------AUGG----UAACGGUUU 5’** | -18.9 |

*N.A.* indicated not analysis.

*The binding sites validated by luciferase reporter assay were highlighted in red.

** The binding sites had been previously reported by using PAR-CLIP.

† Previous reported *BRCA1* binding site of *miR-182-5p* interaction.

The complementarities of the seed region on the binding site were labeled in blue color.


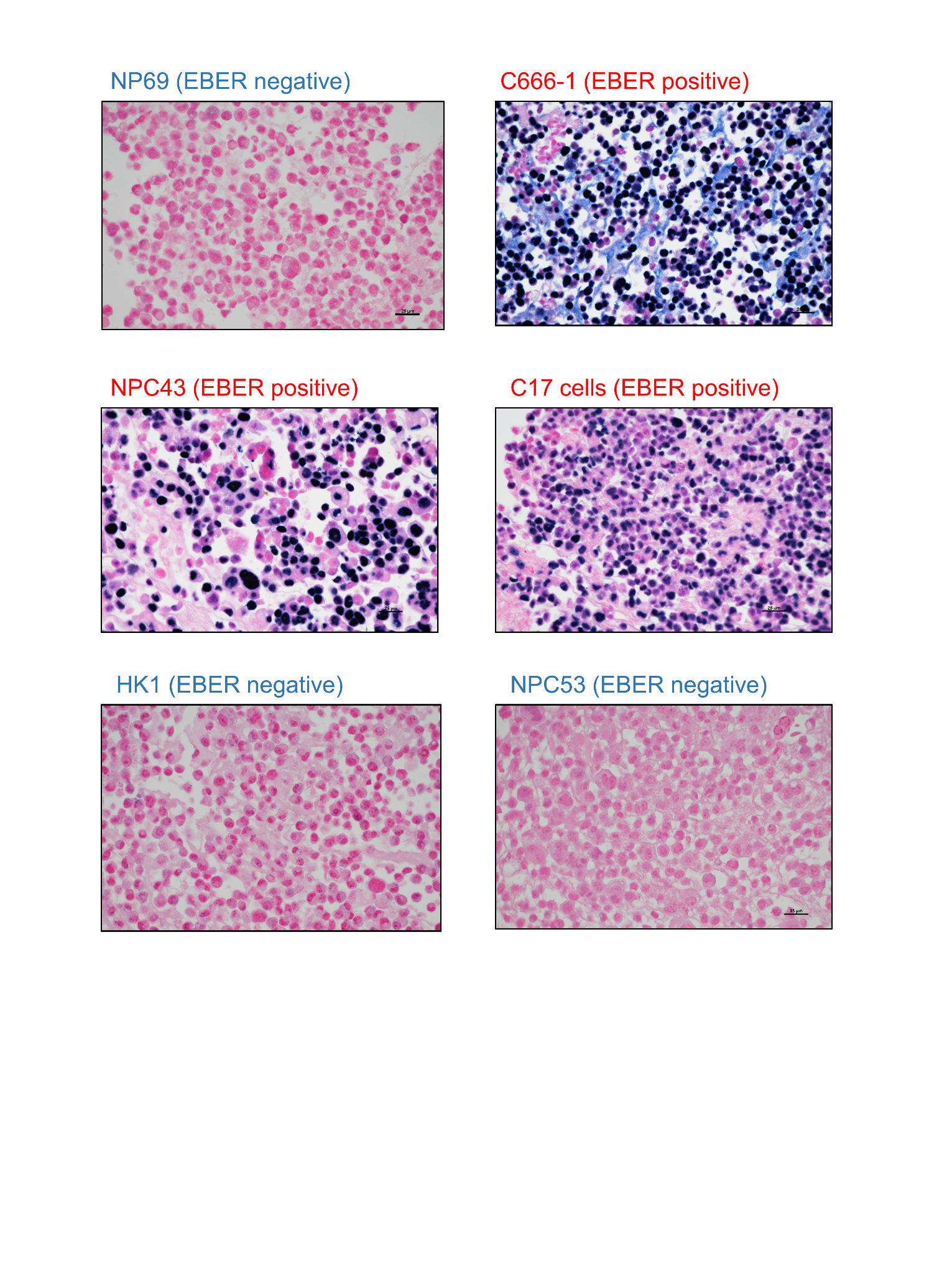


**Figure S1: The EBV status in NPC cell lines.** The presence of EBV in the cell line was confirmed using EBER *in-situ* hybridization with the INFORM EBER probe and ISH iVIEW Blue Detection Kit (Ventana Medical Systems, Tucson, AZ, USA) (original magnification ×400).


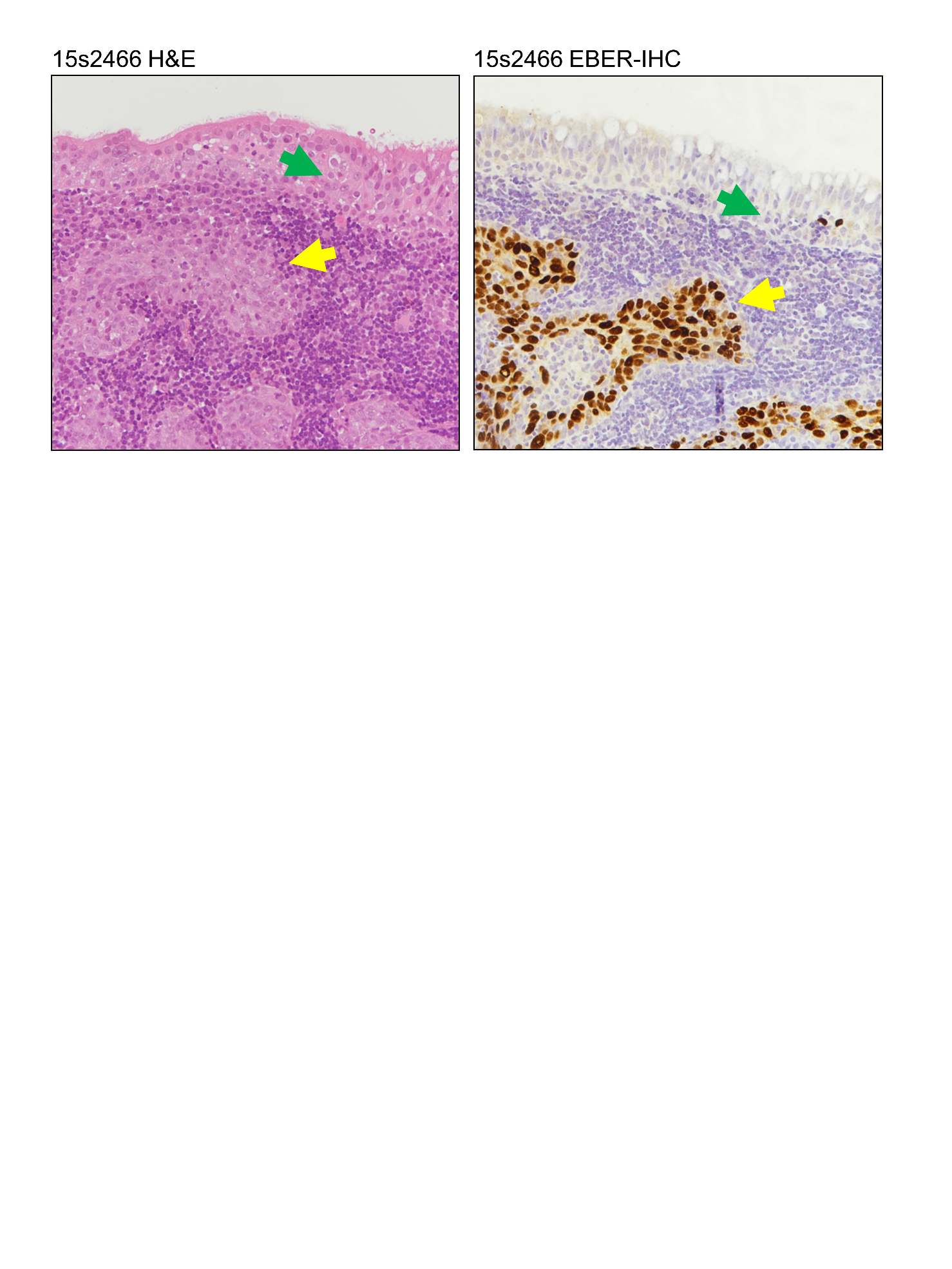


**Figure S2: Detection of EBER in NPC.** The presence of EBV in all NPC specimens was routinely confirmed using EBER *in-situ* hybridization with Bond-polymer Refine Detection kit (Leica Microsystem, Newcastle, UK). The Bond ISH EBER Probe, #BP0589 and the Bond ready-to-use anti-fluorescein-antibody, #AR0833 were used. The H&E staining (left panel) and EBER*-*ISH (right panel) of the representative primary NPC (15s2466) are shown. The nasopharyngeal carcinoma and the normal nasopharyngeal epithelium are indicated by yellow and green arrow, respectively (Original magnification ×400).


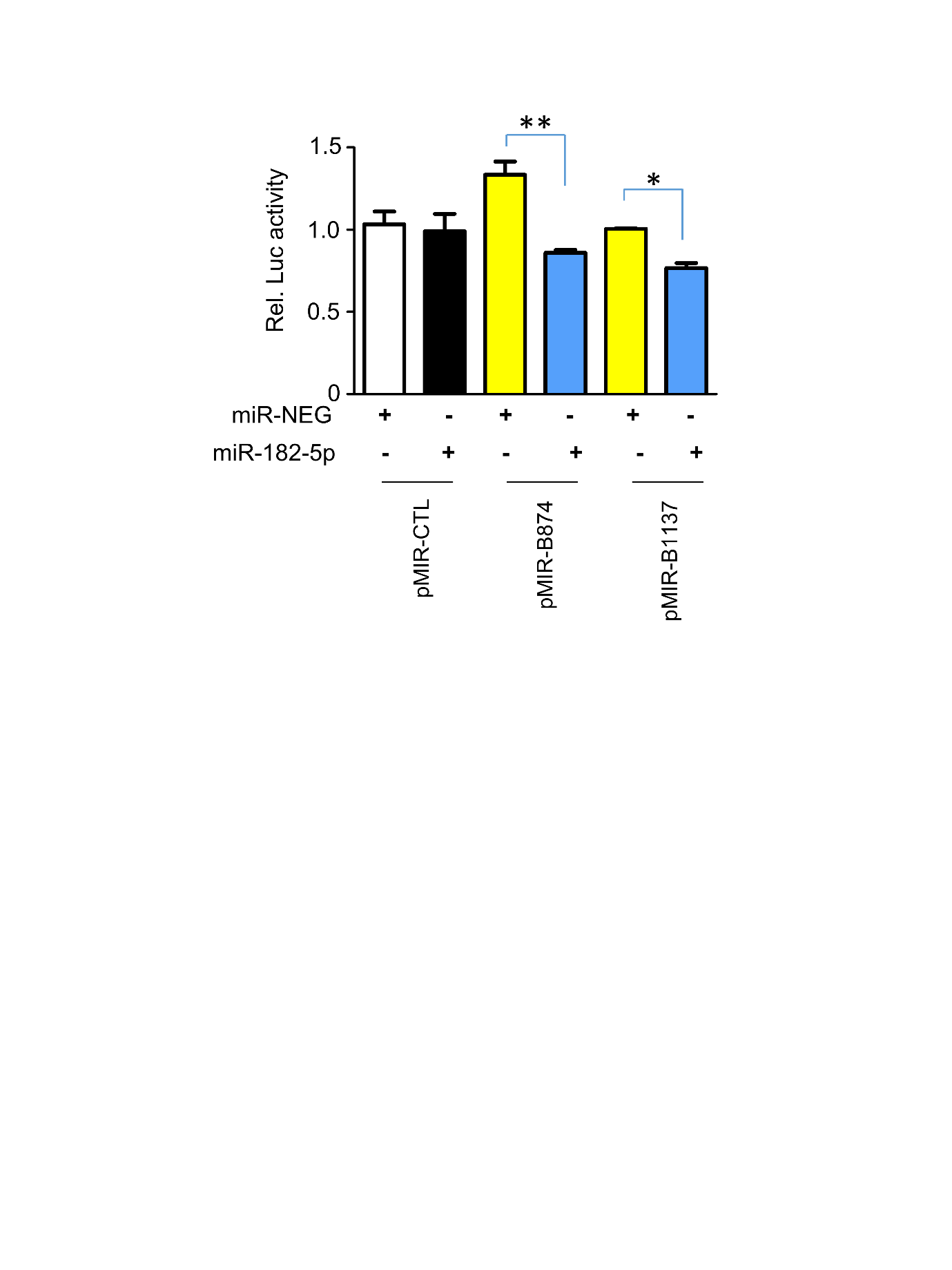


**Figure S3:** **Luciferase reporter assays for miR-182-5p.** The reporter constructs containing putative *miR-182-5p* binding sites (pMIR-B874 and pMIR-B1137) was co-transfected together with *miR-182-5p* or miR-NEG mimics. The firefly luciferase reporter activity was standardised to the *Renilla* luciferase control, and the data shown is the mean + SD from three independent experiments. The result with the co-transfection of miR-NEG and pMIR-CTL was set at 1 for comparison. pMIR-CTL: pMIR-REPORT^TM^ vectors containing unrelated sequences. **P* <0.05*, **P <*0.001.

**
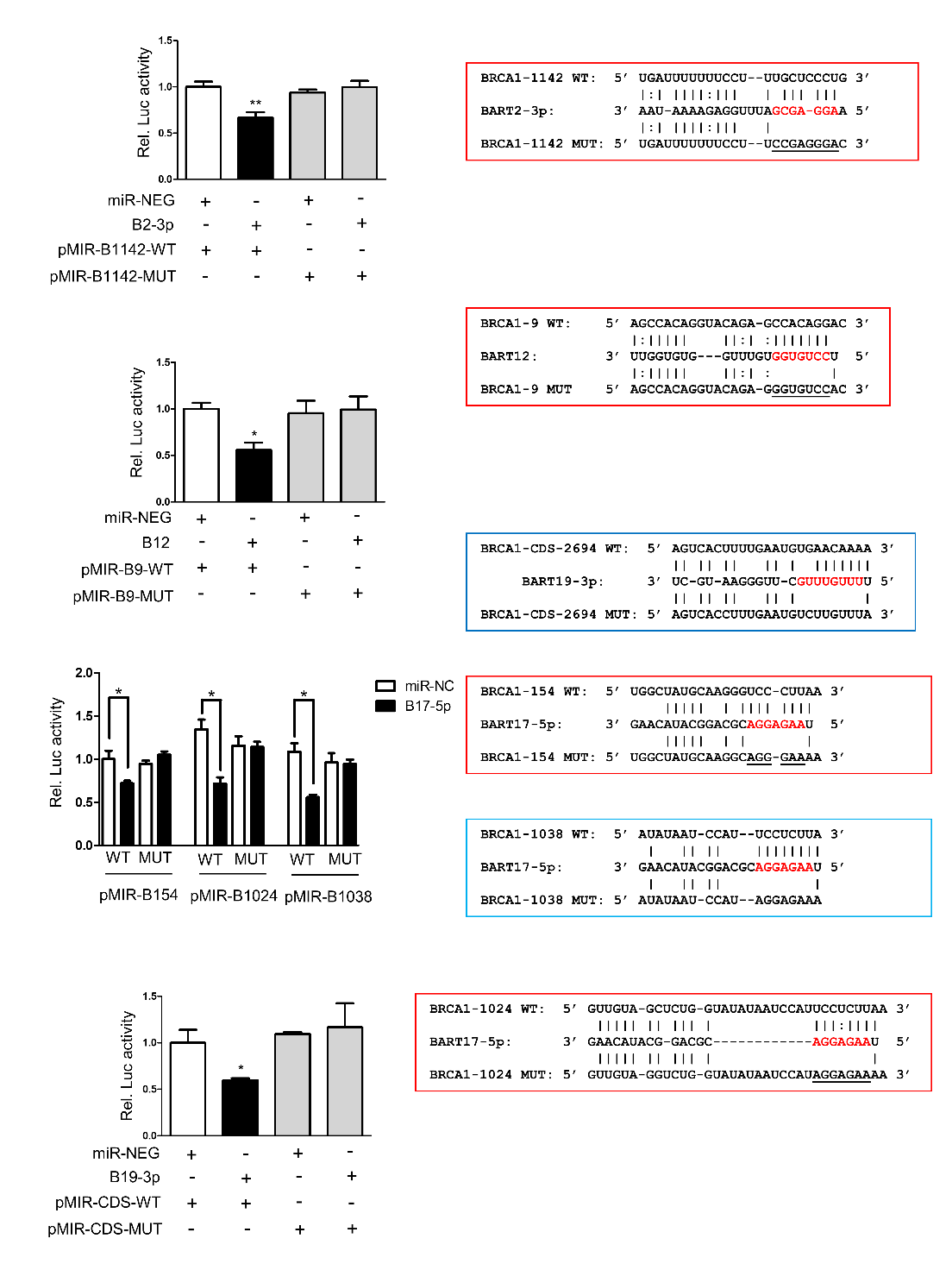
**

**Figure S4: The *miR-BARTs* is not responsive to the putative binding sites, in which the complementarities of the seed region were mutated.** miR-NEG = miRNA negative control mimic; pMIR-B = pMIR-REPORT^TM^ vector harbouring the predicted *miR-BART* binding site, pMIR-B-CDS = predicted binding site on CDS (refer to Table S5), WT = wild type, MUT= binding sites mutated as indicated; B2-3p = BART2-3p; B12 = BART12; B17-5p = BART17-5p; B19-3p = BART19-3p. **P* <0.05*, **P <*0.001.


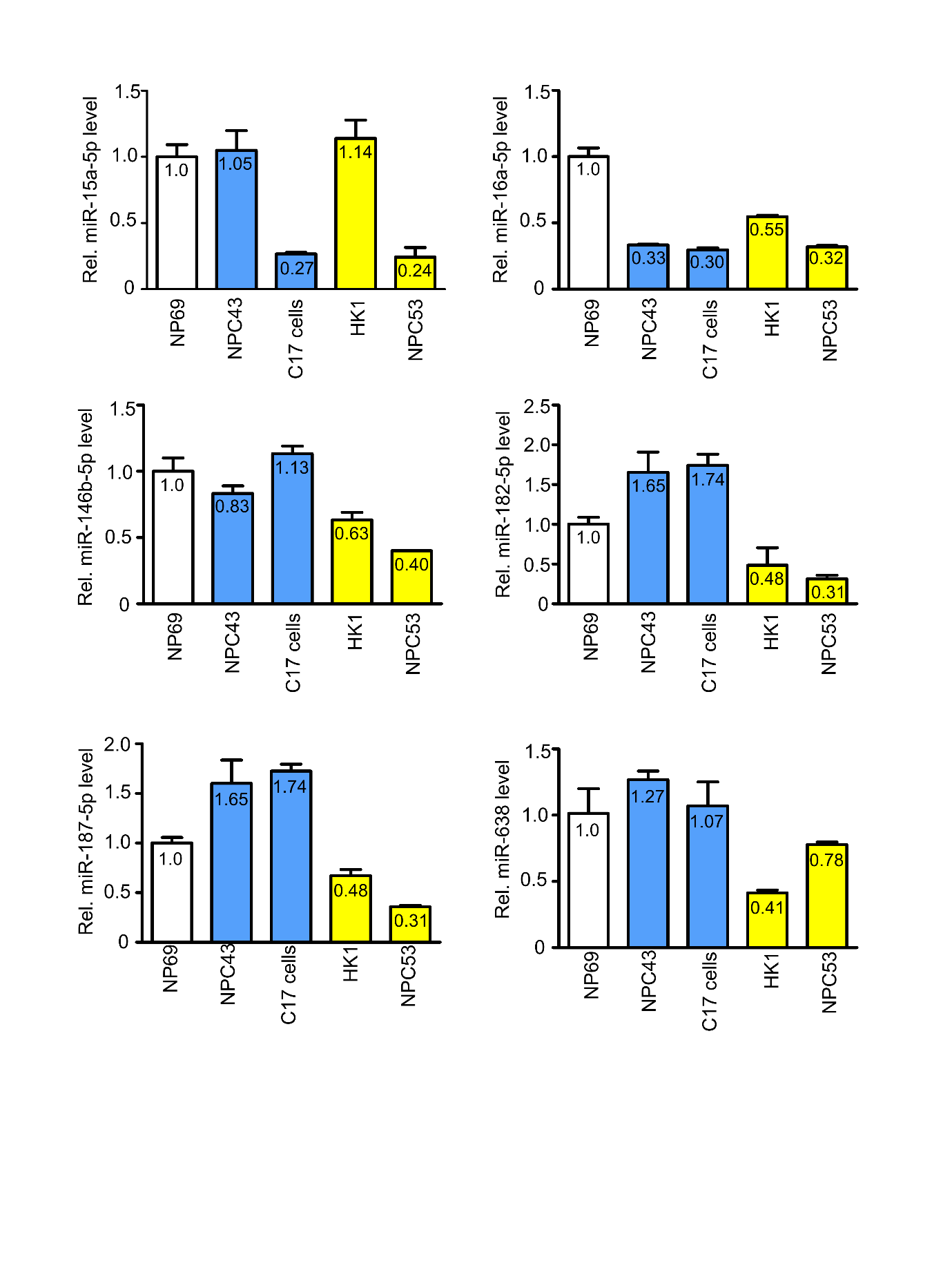


**Figure S5: The expression of previous reported *BRCA1-*repressive miRNAs in NPC cell lines.** The indicated miRNA levels were measured using RT-qPCR. The relative miRNA expressions were normalized with U6, and the values were calculated using the 2^(∆∆-^*^Ct^*^)^ method. Data shown is the mean + SD. White Bar: normal nasopharyngeal epithelial cell line; Blue bar: EBV-positive NPC cell line; Yellow bar: EBV-negative cell lines.


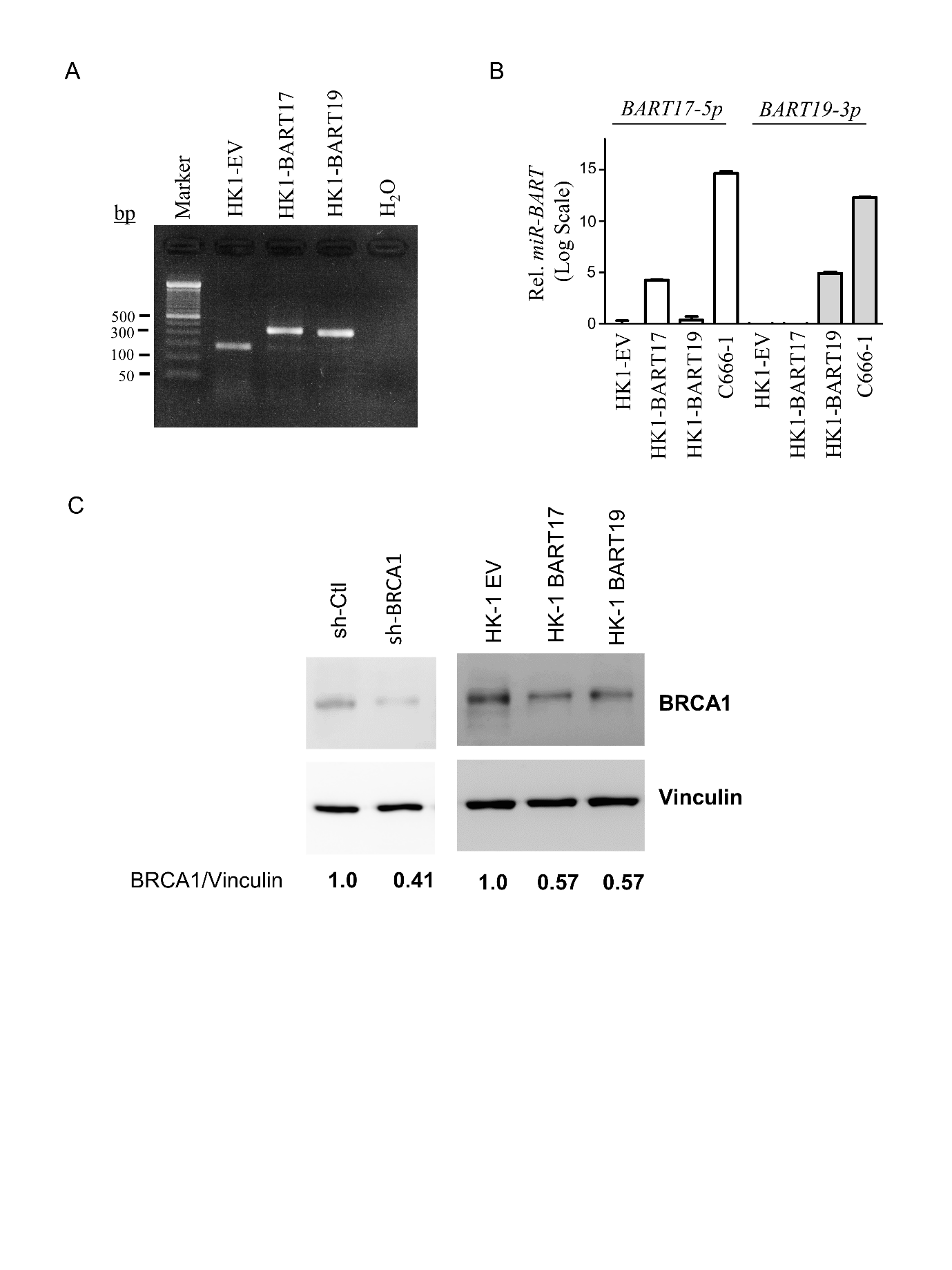


**Figure S6: Downregulation of BRCA1 in the *miR-BARTs-*expressing HK1 stable cells.** (A) The presence of the expressing vector was confirmed using PCR. The genomic DNA of the cells was extracted and served as the PCR template. The expected size of the PCR product from positive clone is 350 bp. (B) The relative expression levels of *BART17-5p* and *BART19-3p* in the stable cells were analyzed using RT-qPCR. The expression levels in C666-1 were included for comparison. (C) The BRCA1 expression in the stable clones was confirmed using the western blot analysis. The HK1 stable cells transiently transfected with sh-BRCA1 was included as positive control.


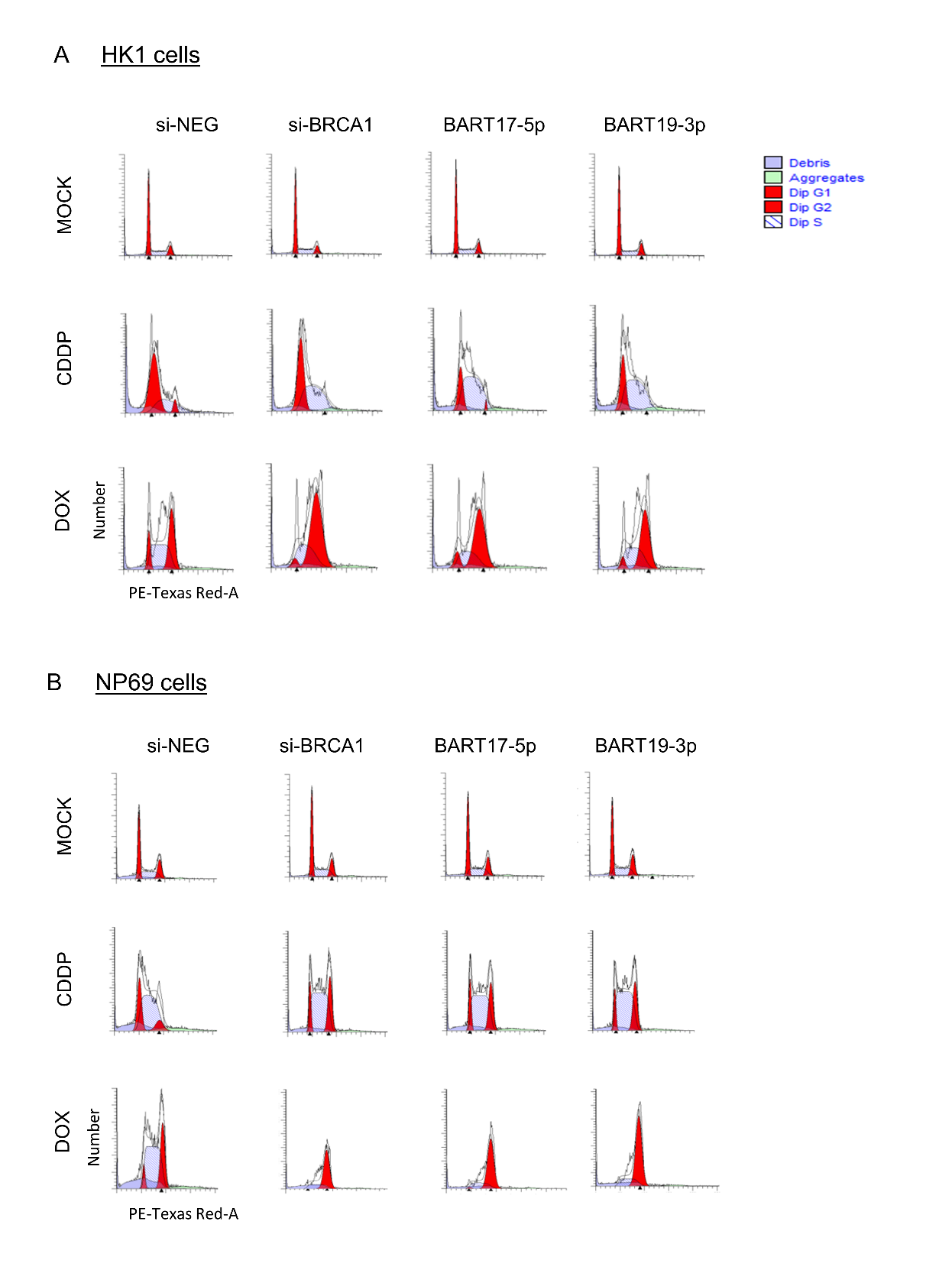


**Figure S7: Transfection of BRCA1-specific si-RNA, *BART17-5p* and *BART19-3p* mimics increased CDDP- and DOX-mediated S phase or G2/M phase cell-cycle arrest.** The transfected HK1 cells and the NP69 cells were incubated with either control buffer (MOCK), cisplatin (CDDP) or doxorubicin (DOX) for 24 hours. Subsequently, the cells were harvested, fixed and their DNA content were analyzed with BD FACSCalibur flow cytometry system with ModFit LT software. The positions of G1-, G2- and S-phases are indicated.


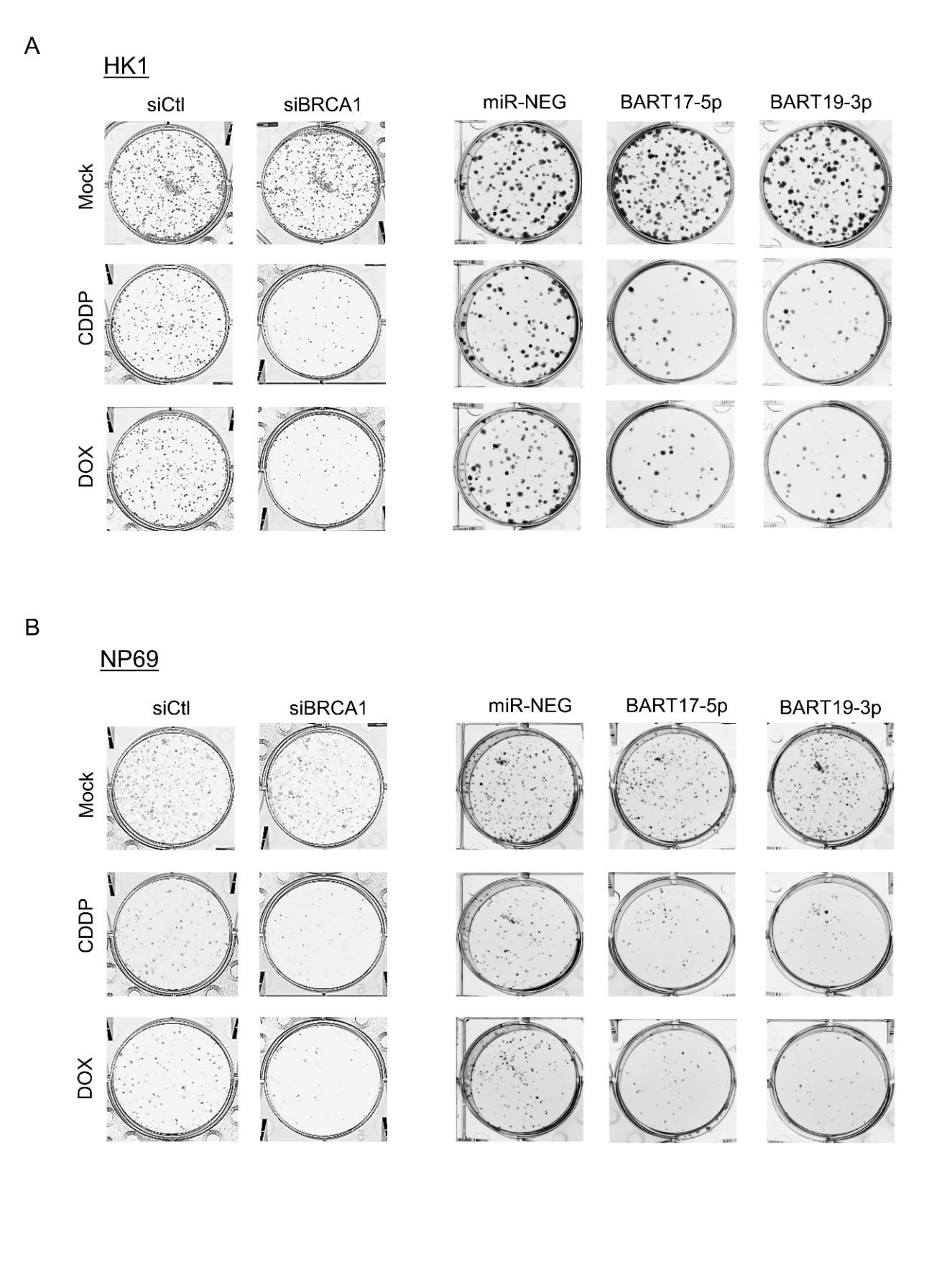


**Figure S8: The long-term clonogenic survival assays.** The BRCA1-specific siRNA, *BART17-5p* or *BART19-3p* mimics transfected cells were incubated with either control buffer (MOCK), cisplatin (CDDP) or doxorubicin (DOX) for 24 hours. Subsequently, 500 cells were seeded into 6-well plate for long term culture in growth medium before staining.


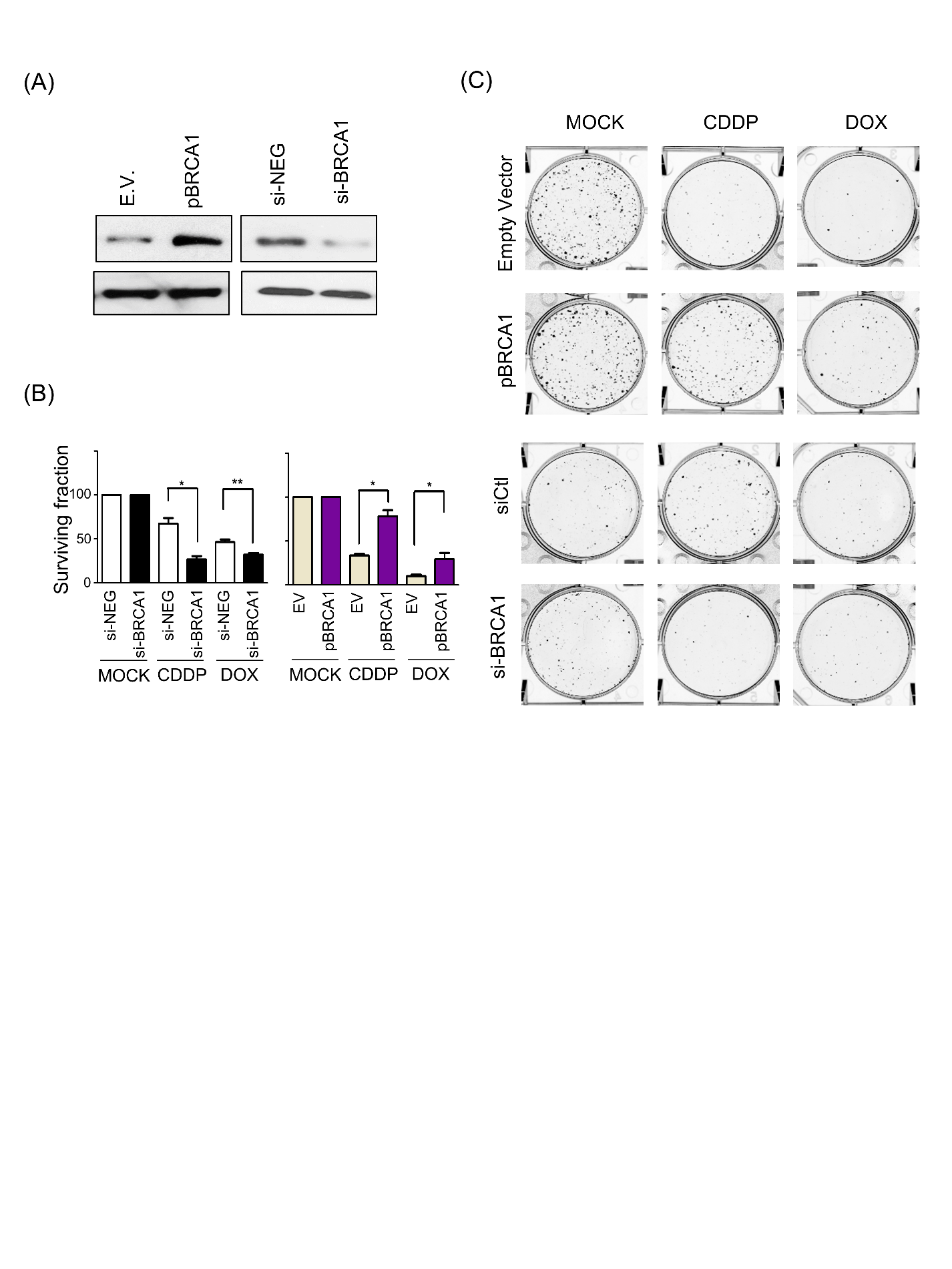


**Figure S9: The cisplatin and doxorubicin sensitivity in C666-1 cells.** (A) The C666-1 cells were transfected with empty vector (E.V.), BRCA1 expression vector (pBRCA1), siRNA control (si-NEG) or BRCA1-specific siRNAs (si-BRCA1) for 24 hours. The BRCA1 expression levels were measured using western blot analysis. (B) and (C) Clonogenic survival assays. The number of colony generated from the mock treatment was set at 100% for comparison. All experiments were performed in triplicate and the Student’s *t-*test was conducted, compared with control transfected cells. **p <* 0.05; ***p <* 0.01.
